# Remittance from migrants reinforces forest recovery for China’s reforestation policy

**DOI:** 10.1371/journal.pone.0296751

**Published:** 2024-06-26

**Authors:** Qi Zhang, Shiqi Tao, Pamela Jagger, Lawrence E. Band, Richard E. Bilsborrow, Zhiqiang Zhang, Qingfeng Huang, Quanfa Zhang, Aaron Moody, Conghe Song

**Affiliations:** 1 Department of Geography and Environment, University of North Carolina at Chapel Hill, Chapel Hill, NC, United States of America; 2 School for Environment and Sustainability, University of Michigan, Ann Arbor, MI, United States of America; 3 Department of Environmental Sciences, University of Virginia, Charlottesville, VA, United States of America; 4 Carolina Population Center, University of North Carolina at Chapel Hill, Chapel Hill, NC, United States of America; 5 School of Soil and Water Conservation, Beijing Forestry University, Beijing, China; 6 School of Forestry and Landscape Architecture, Anhui Agricultural University, Hefei, Anhui, China; 7 Wuhan Botanical Garden, Chinese Academy of Sciences, Wuhan, China; Riphah International University - Lahore Campus, PAKISTAN

## Abstract

Forests play a key role in the mitigation of global warming and provide many other vital ecosystem goods and services. However, as forest continues to vanish at an alarming rate from the surface of the planet, the world desperately needs knowledge on what contributes to forest preservation and restoration. Migration, a hallmark of globalization, is widely recognized as a main driver of forest recovery and poverty alleviation. Here, we show that remittance from migrants reinforces forest recovery that would otherwise be unlikely with mere migration, realizing the additionality of payments for ecosystem services for China’s largest reforestation policy, the Conversion of Cropland to Forest Program (CCFP). Guided by the framework that integrates telecoupling and coupled natural and human systems, we investigate forest-livelihood dynamics under the CCFP through the lens of rural out-migration and remittance using both satellite remote sensing imagery and household survey data in two representative sites of rural China. Results show that payments from the CCFP significantly increases the probability of sending remittance by out-migrants to their origin households. We observe substantial forest regeneration and greening surrounding households receiving remittance but forest decline and browning in proximity to households with migrants but not receiving remittance, as measured by forest coverage and the Enhanced Vegetation Index derived from space-borne remotely sensed data. The primary mechanism is that remittance reduces the reliance of households on natural capital from forests, particularly fuelwood, allowing forests near the households to recover. The shares of the estimated ecological and economic additionality induced by remittance are 2.0% (1.4%∼3.8%) and 9.7% (5.0%∼15.2%), respectively, to the baseline of the reforested areas enrolled in CCFP and the payments received by the participating households. Remittance-facilitated forest regeneration amounts to 12.7% (6.0%∼18.0%) of the total new forest gained during the 2003–2013 in China. Our results demonstrate that remittance constitutes a telecoupling mechanism between rural areas and cities over long distances, influencing the local social-ecological gains that the forest policy intended to stimulate. Thus, supporting remittance-sending migrants in cities can be an effective global warming mitigation strategy.

## 1. Introduction

Forests provide vital ecosystem goods and services [1–4]. In response to calls by the United Nations Framework Convention on Climate Change [5] and the Intergovernmental Science-Policy Platform on Biodiversity and Ecosystem Services [6], numerous national and international conservation funds (e.g., the UN Green Climate Fund) have dedicated substantial resources to slowing, halting, or reversing deforestation and forest degradation. Not every forest conservation investment results in success of net increase in forest cover. Some investments generate mixed and/or even undesirable outcomes [7]. It has been suggested that some programs fail because they target ecologically vulnerable areas where local landholders often bear the brunt of the social and economic costs whereas people far away reap the benefits of the ecosystem goods and services the programs produce [8].

Payments for ecosystem services (PES) have become a widely used forest policy tool to engage forest landholders in strengthening their stewardship of ecosystem services [9,10]. In many cases, a third agency, such as the government, makes the payment (e.g., subsidies) on behalf of the beneficiaries to the landholders who preserve their forests that generate the needed ecosystem goods and services. However, the outcomes of forest condition from PES programs and the livelihoods of the stakeholders are often heterogeneous [11]. PES programs carry a companion goal of poverty alleviation. For these programs to succeed in the long term, it requires that the local population transition to sustainable non-forest dependent livelihoods. Some studies suggest that rural-to-urban labor migration, a hallmark of globalization, can facilitate the progress of forest conservation by reducing poverty and deforestation [12,13]. The main mechanism by which migration facilitates the goals of PES programs is that remittance from migrants substitutes income from extracting forest resources such as timber and fuelwood [14]. Some PES programs can stimulate rural out-migration as a major new livelihood strategy, further amplifying the financial compensations from the PES programs and reinforcing the programs’ outcomes. Remittance connects two distant systems and can be understood as a telecoupling mechanism [15]. Telecoupling represents the coupling between two systems over a long distance, such as soybean trade between China and Brazil influencing farmers’ land use decisions [16,17]. Remittance sent back to origin households by migrants in the cities can modify human-environment interactions in the origin place, for example, by reducing the impact of agricultural failure, reducing the need for forest resource extraction, and/or alleviating pressure on the demand for natural resources [18–20]. Evidence from Nepal, Laos, and Indonesia suggests that households used remittance to buffer shocks associated with agricultural- and forestry-based livelihoods [21].

China has led the global effort in expanding forest cover in recent decades, promoting forest transition nationwide through a series of reforestation and forest-conservation projects [22–24]. Among these initiatives, the Conversion of Cropland to Forest Program (CCFP) is the largest environmental reforestation program in the world based on the PES scheme, in which farmers convert marginal croplands on steep slopes to forests and in turn receive monetary compensation for the ecosystem services (e.g., soil and water conservation) these forests generate. Between 1999 and 2019, the Chinese central government invested a total of US$64 billion on the CCFP, enrolled 41 million households in the program, and successfully created 29.8 million ha of forests through reforestation or afforestation [25]. Following the enrollment of cropland in the CCFP, the demand for farm labor is reduced, freeing labor to shift or diversify livelihoods through off-farm activities. Labor out-migration has been observed as a major labor diversion strategy for the households enrolled in CCFP, and is thought to have the potential of strengthening forest restoration [26–28]. However, the apparent associations between forest increase and migration assumed that migrants are successful in entering off-farm job markets and securing remittance as a major income source for the origin households. Some studies suggest that the effects of migration on forest recovery can be geographically heterogeneous or even unfavorable. Whether and how these mixed outcomes can be explained by migration remains poorly understood.

This study on migrant remittance and forest change resides within the conceptual framework that incorporates telecoupling relationships and coupled natural and human (CNH) systems. Research in CNH systems is situated at the core of sustainability science [29], as it addresses questions pertaining to the intricate and intimate interconnections between people and their environment [30–32]. Core characteristics of CNH systems include reciprocal feedbacks, heterogeneity, and nonlinearity. CNH dynamics involve the flow of capital in difference forms, such as natural capital (e.g., firewood harvested from forest) and human capital (e.g., farm labor as input for land cultivation) [33,34]. Environmental and/or development policies targeting ecologically sensitive areas need to consider such dynamics from a CNH perspective in order to achieve social-ecological sustainability [35]. One key issue is what is known as the “poverty trap”, within which resource users rely so heavily on the environment that their continued usage causes environmental degradation and natural resource depletion, making them unable to escape the poverty on their own without external intervention [36]. Policymakers need to understand such human-environment interactions while implementing environmental policies in order to avoid policy failures. Telecoupling, in the context of CNH systems, involve socioeconomic and environmental interrelationships between distant CNH systems, including flows of information, energy, matter, people, organisms, as well as financial capital and goods and products (Hull and Liu et al., 2018). The strength and longevity of connections between CNH systems as well as their local impacts do not necessarily diminish as a function of geographical distance. For instance, agricultural land-use change at the local level in Brazil and China depends on the international trade of soybean between the two countries [16,17,37]. In our case, forest and people in the rural areas represent the local CNH systems, while the far-away cities where migrants earn incomes represent the distant CNH systems. The two CNH systems are connected via rural out-migration. The rural CNH systems send out migrants to and receive remittance from the distant CNH systems, which in turn receive migrants as labor from and send out the remittance to the rural CNH systems. Thus, we hypothesize that remittance from migrants in distant CNH systems is a major telecoupling mechanism that plays a key role in altering the social-ecological dynamics in the local CNH systems.

In this study, we adopted a multilevel analysis and performed quasi-experimental approaches to investigate the role of remittance in forest-livelihood dynamics under China’s CCFP, aiming at conceptual advancement for the framework of telecoupling and CNH systems. Specifically, we collected in-depth socioeconomic and demographic data through comprehensive household surveys in two rural areas of China during 2014–2015, and integrated data analysis with inputs from satellite remote sensing. We traced the migration destinations from our study sites to places all over the country and depicted the migration and remittance flows that feature the telecoupling relationships between local rural CNH systems and distal urban CNH systems of cities. Participation in the CCFP in the rural CNH system influenced household labor allocation for migration. Our research aim is to examine the mediating role of remittance from migrants in forest regeneration and poverty alleviation as a result of the CCFP. Three main objectives are: i) to investigate the effects of the CCFP on remittance sent by migrants to their origin households, ii) to evaluate the impacts of remittance on forest dynamic surrounding the origin households, and iii) to estimate socioeconomic and ecological additionalities from the CCFP mediated by remittance, namely the proportions of CCFP-stimulated remittance in total CCFP investment and remittance-induced new forest area in the baseline of CCFP forest area, respectively.

## 2. Materials and methods

### 2.1. Household and land surveys

We collected socioeconomic-demographic and spatial data from the two study sites in rural China where CCFP was implemented (**S1 Text and S1 Fig**). We carried out two parallel household surveys in Tiantangzhai (TTZ) of Anhui Province and Jichang & Checheng (J&C) of Shanxi Province during the summers of 2014 and 2015, respectively. Before the surveys, a two-level stratified disproportionate sampling scheme was adopted to select comparable number of CCFP-participating households and non-participants in the sample [38,39]. The questionnaire covers topics including demographic information of all household members and migrants, remittance from migrants, areas of cropland enrolled in CCFP, firewood collected from forests, among others. Graduate students from local universities were recruited and trained for two weeks as the interviewers for the surveys. For each interviewed household, we recorded the geographic coordinates at the house location with a handheld Global Positioning System unit. Topographic conditions (e.g., elevation and slope) at the house location were derived based on the digital elevation model. The surveys obtained complete data for a total of 2,905 individuals from 731 households from the two sites. We identified 1,994 individuals aged 15–59 and then selected a subsample of 767 individuals (38.5%) from 458 households who had migrated outside the local county for at least six consecutive months and still lived away from home at the survey time, termed *migrants* here. Of the sampled migrants, 36% sent remittances to their origin households during the 12 months prior to the survey time (**Table 1**). Through household surveys, we obtained information about CCFP participation and cropland use, recording area of cropland enrolled in the CCFP program and area of cropland managed by the households. Given the fixed CCFP payment rate [40], the amount of payment received by a participating household can be calculated based on the area of the enrolled cropland.

**Table 1:** Statistical summary of remittance sent by migrants and PES payments of CCFP.

Category	All observations	Observations (CCFP = 1)	Observations (CCFP = 0)
***Individual level***	All individual migrants	Migrants from CCFP-participating households	Migrants from non-participating households
Whether sent remittance			
Total	767	444	323
Yes	277 (36.1%)	160 (36.0%)	117 (36.2%)
No	490 (63.9%)	284 (64.0%)	206 (63.8%)
Remittance amount sent			
Mean (SD) (1,000 Yuan)	16.1 (21.9)	19.1 (25.9)	15.5 (21.0)
***Household level***	All households	CCFP-participating households	Non-participating households
Whether received remittance			
Total	458	261	197
Yes	233 (50.9%)	134 (51.3%)	99 (50.3%)
No	225 (49.1%)	127 (48.7%)	98 (49.7%)
Remittance amount received			
Mean (SD) (1,000 Yuan)	19.8 (23.4)	21.9 (29.0)	19.3 (22.1)

Note: Mean and standard deviation (SD) are estimated with sample weights. T-tests show that 1) the amount of remittance sent by migrants from CCFP households is significantly higher than that by migrants from non-participating households (t = 3.49, *p* = 0.000), 2) the summed amount of remittance received by CCFP-participating households are significantly higher than those by non-participating households (t = 2.15, *p* = 0.031). The unit of remittance amount is 1,000Yuan; US$1 ≈ 6.22 Yuan (2014–2015).

### 2.2. Multilevel analysis of CCFP payments and remittances with mixed-effects models

Guided by theoretical frameworks of sustainable livelihoods [41], telecoupling [8] and social-ecological systems [35], we hypothesize that the decision of sending remittance and the amount of remittance sent by an individual migrant are determined by factors across multiple levels. Personal attributes at the individual level include gender, age, education, and whether the migrant lives in cities outside the province, indicating the person’s capability of pursuing economic opportunities. Whether an origin household receives remittance or not depends on natural capital (e.g., land endowment), physical assets (e.g., house condition), human capital (e.g., household head education), financial capital (e.g., cash received from CCFP), and the geographic conditions at the house location (e.g., accessibility to the local market). Community-level factors may also influence household livelihood strategies through social connections or neighborhood effects. Therefore, we include a set of explanatory variables at the individual, household, and community levels (**S1 Table**). We derived the cumulative inflation-adjusted amount of CCFP payment to individual households during the migration years as the variable for CCFP participation. This variable captures both the participation status of the PES program with cropland enrollment for reforestation but also the financial compensation to cover the opportunity costs of forgoing the income from the cropland enrolled in CCFP. Preliminary statistical analysis indicates that CCFP payment does not significantly correlate with the other explanatory variables (**S3 Fig**).

We performed a multilevel analysis to model remittance influenced by multiple factors including the CCFP. Multilevel regression models can simultaneously capture both fixed effects of the explanatory variables and the random effects at different levels, making the estimated coefficients robust to bias from group variances at higher levels when using hierarchically structured datasets [42,43]. We first fitted a mixed-effects logistic regression model to examine the probability of sending remittance by an individual migrant. Let individual *i* (*i* = 1, 2, …, *n*_*c*_) reside within resident group *c* (*c* = 1, 2, …, *C*), the two-level mixed-effects logistic model can be specified as:

$$Pr\left(y_{ic}=1|x_{ic},u_{c}\right)=L\left(x_{ic}\beta +z_{ic}u_{c}+\varepsilon_{ic}\right)$$ (1)

Where *y*_*ic*_ is the binary response variable with 1 denoting individual migrants sending remittance and 0 otherwise. In this study; ***x***_*ic*_ is a 1 × *p* vector of explanatory variables for estimating a *p* × 1 vector of fixed effects, *β*; ***z***_*ic*_ is a 1 × *q* vector of explanatory variables corresponding to the *q* × 1 vector of random effects, ***u***_***c***_, which are *C* realizations from a multivariate normal distribution *N*^*m*^(0, *Σ*) with *Σ* representing the summarized variance components of the random effects; *L*(.) is the function of the logistic cumulative distribution that maps the linear predictor to the probability of a positive response, i.e., sending remittance; *L*(.) = *e*^*ν*^/(1+*e*^*ν*^) while *e* is the base of the natural logarithm and *ν* is the linear component; the error component *ε*_*ic*_ has a distribution *N*^*l*^(0, *π*^*2*^/3) as logistic, independent of ***u***_***c***_.

We then fitted a multilevel mixed-effect linear regression model to examine the *amount* of remittance sent by an individual migrant. The amount of remittance across the households has a right skewed distribution and was hence logarithmically transformed. The two-level mixed-effects linear model can be specified as:

y=Xβ+Zu+τ (2)

Where **y** is a *n* × 1 vector of the response variable containing continuous values that indicate the amounts of remittance sent by the migrants; **X** is a *n* × *p* matrix of explanatory variables for estimating a *p* × 1 vector of the fixed effects, ***β***, and the component ***Xβ*** is the fixed part of the model; **Z** is a *n* × *q* matrix of explanatory variables corresponding to a *q* × 1 vector of the random effects, *u*; *τ* is a *n* × *1* vector of the overall residual, following a multivariate normal distribution, denoted as *τ*∼*N*^*m*^(0, *σ*_***τ***_^2^R). The random part of the model is made up of two components, noted as ***Zu*** + *τ*, where ***u*** comprises the variance-covariance matrix Ω orthogonal to *τ*, so that Cov(*u*, *τ*) = 0. Here, we estimated the specified models by considering the random intercept, which sets *z* (or *Z*) as the scalar 1.

### 2.3. Matching households receiving remittance with those not receiving remittance

We conducted propensity matching for households with migrants receiving remittances to households with migrants not receiving remittances before estimating the effects of remittance on forest change with regression analysis. Since our aim was to examine forest dynamics surrounding the house locations at the household level, we aggregated the hierarchical data on individual-level remittance to household-level variables, including the total amount of remittance received by the origin households and the average amount of remittance per migrant in a household. We derived a binary variable to indicate whether the household receives any remittance or not. Households receiving remittance constitute the treated group, while those not receiving remittance the control group. Among the 458 households, 51% received remittances (**Table 1**), providing a balanced sample size for treated and control groups.

We measured outcomes as the changes of forest cover and forest greenness surrounding the household within circular buffers around each house location. Forest dynamics involve spatial properties (e.g., impact range), so we performed sensitivity analysis of the effects by generating buffers with a series of radii from 25 to 200 m, at an increment of 25 m. We calculated two forest-related measures within each buffer based on data layers derived from satellite images [44], including the proportion of forest cover and the mean Enhanced Vegetation Index (EVI) of the forested areas. EVI is an index of vegetation greenness based on satellite remote sensing data; the EVI value ranges from -1 to 1 with dense and healthy vegetation have high values [45]. Thus, the outcome variables are the differences of the two indicators between the survey year (i.e., 2013 in TTZ and 2014 in J&C) and the starting year of CCFP implementation (i.e., 2002), noted as *ΔForest* and *ΔEVI*, respectively. Within a buffer, the equations for deriving the outcome variables can be formulated as follows.


$$\Delta Forest=\frac{1}{n}\left(\sum_{i=1}^{n}K_{i1}-\sum_{i=1}^{n}K_{i0}\right)$$ (3)

$$\Delta EVI=\frac{\sum_{i=1}^{n}K_{i1}\eta_{i1}}{\sum_{i=1}^{n}K_{i1}}-\frac{\sum_{i=1}^{n}K_{i0}\eta_{i0}}{\sum_{i=1}^{n}K_{i0}}$$ (4)

Where *n* is the total number of grids within the buffer circle; *i* represents a grid within the buffer; *K*_*i1*_ and *K*_*i0*_ are indicators representing whether the grid is classified as forest (*K* = 1) or non-forest (*K* = 0) in the survey time (2014 for J&C, 2013 for TTZ) and the CCFP starting time (2002), respectively; *η*_*i1*_ and *η*_*i0*_ are EVI values at grid *i* in the survey year and CCFP starting year, respectively. Grids are included if at least 0.5% of the grid is in the buffer and their weight is the fraction of the grid overlapping with the buffer.

By considering major differences in regional context, topographical condition, accessibility to market, endowment of natural capital (e.g., cropland area) and physical capital (e.g., farm tools), we controlled for several covariates that may confound the effects of receiving remittance on forest dynamic (**S6 Table**). We fitted a logistic regression model to predict the propensity score (i.e., probability) [46] for a household receiving remittance from migrants. The accuracy of the model is 70.7% (**S4 Fig**, upper panel), indicating a relatively high degree of separability between the two groups and the need for matching. We used the one-to-one matching procedure [46] to match the household receiving remittance with the closest score to each of the households without remittance due to the slightly larger sample size of the former. The matched sample consisted of 450 matched households (or 225 pairs), among which the treated households with multiple matches were weighted by the inverse of the number of times a household had a match. The mean value of score difference after matching was not significantly different from zero (t = -0.42, *p* = 0.68), suggesting a satisfactory performance of the propensity score matching (**S4 Fig**, lower panel). Comparison of covariates showed that the effects of all the hypothesized confounders have been reduced to a satisfactory degree after matching (**S6 Table**). The matching caliper defines the closeness of the scores between two households from the two groups to be matched, with a smaller caliper suggesting closer scores. Sensitivity analysis to the matching caliper demonstrated that such effects are robust at relatively large thresholds with sufficiently retained households (**S2 Text and S6 Fig**).

### 2.4. Estimating average treatment effects and remittance-forest associations

We adopted pairwise bootstrapping to estimate the average treatment effect of remittance on forest change and explored the remittance-forest association. Bootstrapping mimics the sampling process by randomly selecting a subset of the sample with replacement and hence can quantify the uncertainty of the estimators, such as regression coefficients and significance levels [47]. We processed 1,000 repetitions of resampling and randomly select 60% of the 225 pairs of matched households at each repetition. The sample size proportion of 60% was empirically determined to balance between assessing the consistency level of the effects and maintaining sufficient sub-sample size for meaningful statistical inference [48].

In line with our main hypothesis, we tested three specific hypotheses: i) the difference of forest changes between households receiving remittance and those not receiving remittance is significantly positive (H1: Treated—Control > 0), ii) forest change surrounding households receiving remittance is significantly higher than zero (H2: Treated > 0), and iii) forest change surrounding households without remittance is not significantly different from zero or significantly below zero (H3: Control ≤ 0). A series of *t*-tests were performed to test these hypotheses within the varying sized circular buffers. The percentage of occurrence of significant difference (*p* < 0.05) among the 1,000 repetitions was recorded to measure the consistency level. Next, we used ordinary least-squares regression to explore the associations between forest change and remittance indicators. Of the repetitions, similarly, we computed the mean values for the estimators, namely the regression coefficients, as well as the percentage of occurrence of significant effects at the 5% significance level.

The matching process itself did not consider the spillover effects across spatial units [49]. In this case, a household may extract forest resources in areas overlapping with the surrounding area of other households and vice versa. To address this issue, we divided households that form spatial clusters into 63 resident groups and aggregated the total amount of remittance received from migrants for each group (**S7 Fig**). Since household clusters are polygons with areal attributes, we generated ring buffers at 0–100 m and 100–200 m surrounding the boundary of a resident group and derived the same indicators of forest change as described above within the ring buffers, in addition to the group polygon area within the boundary. We generated scatterplots of the indicators of remittance and forest dynamic to evaluate the robustness of the estimated effects.

### 2.5. Mechanism analysis of households influenced by remittance

Rural households, particularly those living in proximity to forests, extracted forest resources such as fuelwood to meet their livelihood needs [39]. Thus, we hypothesize that the mechanism of remittance influencing forest dynamic was related to the reliance on forest resources for livelihood support. To examine this mechanism, we derived indicators on forest livelihoods including the share of fuelwood in total energy use, raising livestock, and inputs for extracting resources (in TTZ only). The share of fuelwood use was measured as the percentage of the estimated value of total used fuelwood in the sum of the fuelwood value and cost of gas (natural gas and/or liquefied petroleum gas) per year, where the value of fuelwood was calculated by multiplying the estimated weight of fuelwood by the unit price (0.4 Yuan/kg) based on the information collected during the household surveys. We statistically tested the difference of the indicators between households receiving remittance and those with migrants but not receiving remittance, as well as quantify the relationships between the remittance amount and the indicator values. In addition, to reflect the households’ stages of energy transition, we group households into three categories along the energy ladder: 1) using fuelwood (and/or coal) as the only or main energy source; 2) using about half fuelwood (and/or coal) and half modern fuels (e.g., gas and electricity) for energy; 3) using only or primarily modern fuels (e.g., natural gas), based on the survey data, and used the χ^2^ test to measure the difference in composition between the two household groups.

### 2.6. Estimating socioeconomic and ecological additionalities of CCFP mediated by remittance

Following the analyses of the remittance-forest associations, we estimate the additional socioeconomic and ecological effects of the PES investment for forest conservation that are mediated by remittance as follows.


$$G_{Econ}\text{\%}=\frac{\sum_{i=1}^{N}10^{\hat{\beta }\left(M_{i}P_{i}/1000\right)}}{\sum_{h=1}^{H}P_{h}}\times 100\text{\%}$$ (5)

$$G_{Ecol}\text{\%}=\frac{\sum_{h=1}^{H}\hat{\gamma }L_{h}\left(P_{i},M_{i},\hat{\beta }|i\in S_{h}\right)A}{\sum_{h=1}^{H}P_{h}/\lambda }\times 100\text{\%}$$ (6)

Where *G*_*Econ*_% is the percentage of socioeconomic gain due to the PES investment that is mediated by remittance; *i* represents an individual out-migrant, and *h* represents a household; *N* is the size of the individual sample, while *H* is the size of the aggregated household sample; $\hat{\beta }$ is the estimated coefficient based on the multilevel mixed-effect linear regression model (0.099 ± 0.026 in Model 3); *M*_*i*_ is the migration period for individual *i* (unit: year); *P*_*i*_ is the cumulative inflation-adjusted amount of CCFP payment corresponding to individual *i* (unit: Yuan/year) from household *h*; *P*_*h*_ is the CCFP payment corresponding to household *h* (unit: Yuan/year). The uncertainty level is estimated by one standard deviation (${\hat{\sigma }}_{\beta }$) range of the estimated coefficient, i.e., $\hat{\beta }\pm {\hat{\sigma }}_{\beta }$. One household has an estimated remittance amount outside the model predictable range (z-score of 21.05 among households receiving remittance) and is hence excluded for the estimation. Meanwhile, *G*_*Ecol*_% is the percentage of ecological gain due to the PES investment that is mediated by remittance; $\hat{\gamma }$ is the estimated coefficient of linear regression between remittance (logarithm) and forest-cover change (0.011 ± 0.005); *L*_*h*_*(*.*)* is the function of aggregating individual-level remittances to the sum of remittances received by household *h* (logarithm), where individual *i* belongs to the individual set of households *h*, *S*_*h*_; *A* is the area of the circular buffer surrounding the household; *λ* is the PES payment rate (0.135 Yuan/year/m^2^ for J&C; 0.1875 Yuan/year/m^2^ for TTZ). Uncertainty from estimating effects of CCFP payments on remittance can propagate to the effect of remittance on forest dynamic. Thus, we estimate the uncertainty degree by considering the multiplications of the lower (or upper) limits of both estimators.

## 3. Results

### 3.1. Telecoupling networks of migration and remittance flows

**Fig 1:**
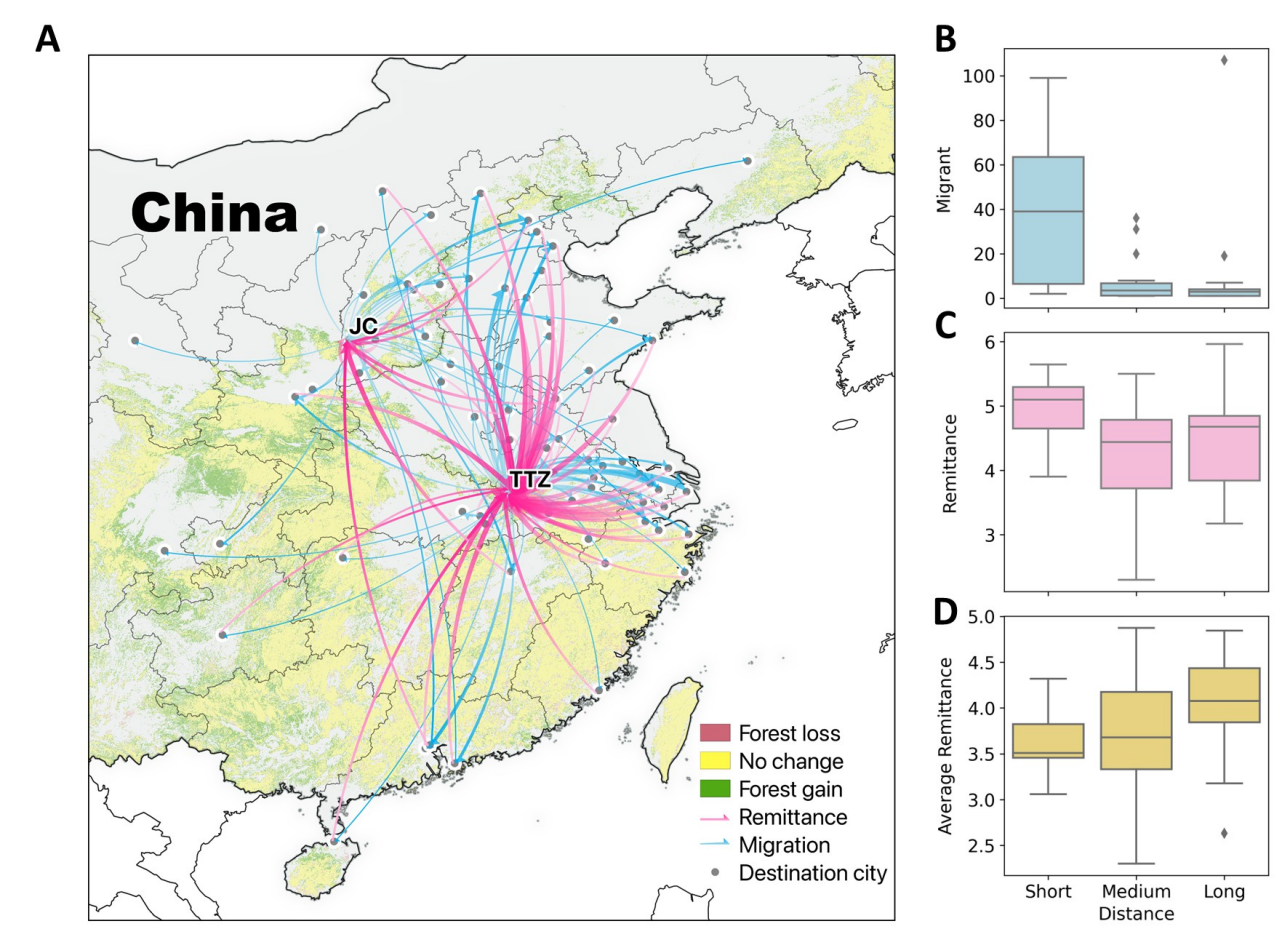
Telecoupling relationships between rural and urban areas through migration and remittance flows. (A) Map of migration outflows and remittance inflows between study sites and distal cities over the country. A wider arrow indicates a larger volume in migration (number of migrants) or remittance (amount). A higher opacity level of an arrow indicates a longer distance. JC and TTZ represent two study sites. (B) Distributions of migrant number, (C) remittance, and (D) remittance per migrant by distance ranges at the provincial level. The ranges of distance (logarithm) are 1.7∼2.2 (short), 2.2∼2.7 (medium), 2.7∼3.2 (long) at the city level. ANOVA tests were used to test difference among short, medium and long distant migration (Number of migrants: F = 7.80, *p* < 0.01; Amount of remittance: F = 2.12, *p* = 0.13; Remittance per migrant: F = 2.35, *p* < 0.11. Data source for forest changes during 2000–2015 is Global Forest Cover Change by Sexton et al. **[53]**. A pixel is defined as forested where the fraction of forest cover is greater or equal to 30%, following the criteria by Hansen et al. **[54]**.

We trace the migration destination for each out-migrant from the surveyed households in our study sites during the period when China experienced significant forest gains, which help develop the flow map of migration and remittance over the country (**Figs 1 and S2**). The networks of migrant outflows and remittance inflows can be interpreted by two attributes, including the distance between origin and destination places and the intensity measured in remittance amounts per migrant from the same destination. About 63% (55 out of 87 cities) of the reported destination cities have witnessed remittance sent by the migrants, while the percentage is 66.7% (24 out of 36 provinces) at the provincial level when aggregating the amount of remittance for provinces. The longest city-level telecoupling distances are found in Guangzhou (Guangdong) and Haikou (Hainan) in Southern China from the semi-arid J&C and subtropical TTZ study sites, respectively (**Fig 1A**). Destinations in relatively short distances observe larger volumes of migrants as well as significantly higher amounts of remittance (**Fig 1B** and **1C**). In contrast, the amount of remittance per migrant is larger for longer-distance migrants, although the difference is not statistically significant (**Fig 1D**). Interestingly, sending remittance by migrants is not weakened by the increased migration distance. Such findings are consistent when setting the destinations as the capital cities at the provincial level (**S2 Text and S2 Fig**). These observed patterns, as expected from theoretical models and empirical understanding [50–52], demonstrate strong telecoupling relationships even over long distances.

### 3.2. Effect of CCFP on remittance by migrants

Based on our household survey, 36% of the 767 individual migrants have sent remittances to their origin households (**Table 1**). The remittance amount sent by migrants from CCFP-participating households is significantly higher than that by migrants from non-participant households (t = 3.49, *p* < 0.01), with the former 19,100 Yuan ($US3,071) per year and the latter 15,500 ($US2,492) per year on average. Aggregating to the household level, 51% of the total 458 households receive remittances from migrants. Meanwhile, CCFP-participating households and non-participants receive 21,900 Yuan ($US3,521) and 19,300 Yuan ($US3,103), respectively, their difference being statistically significant (t = 2.15, *p* < 0.05).

**Fig 2:**
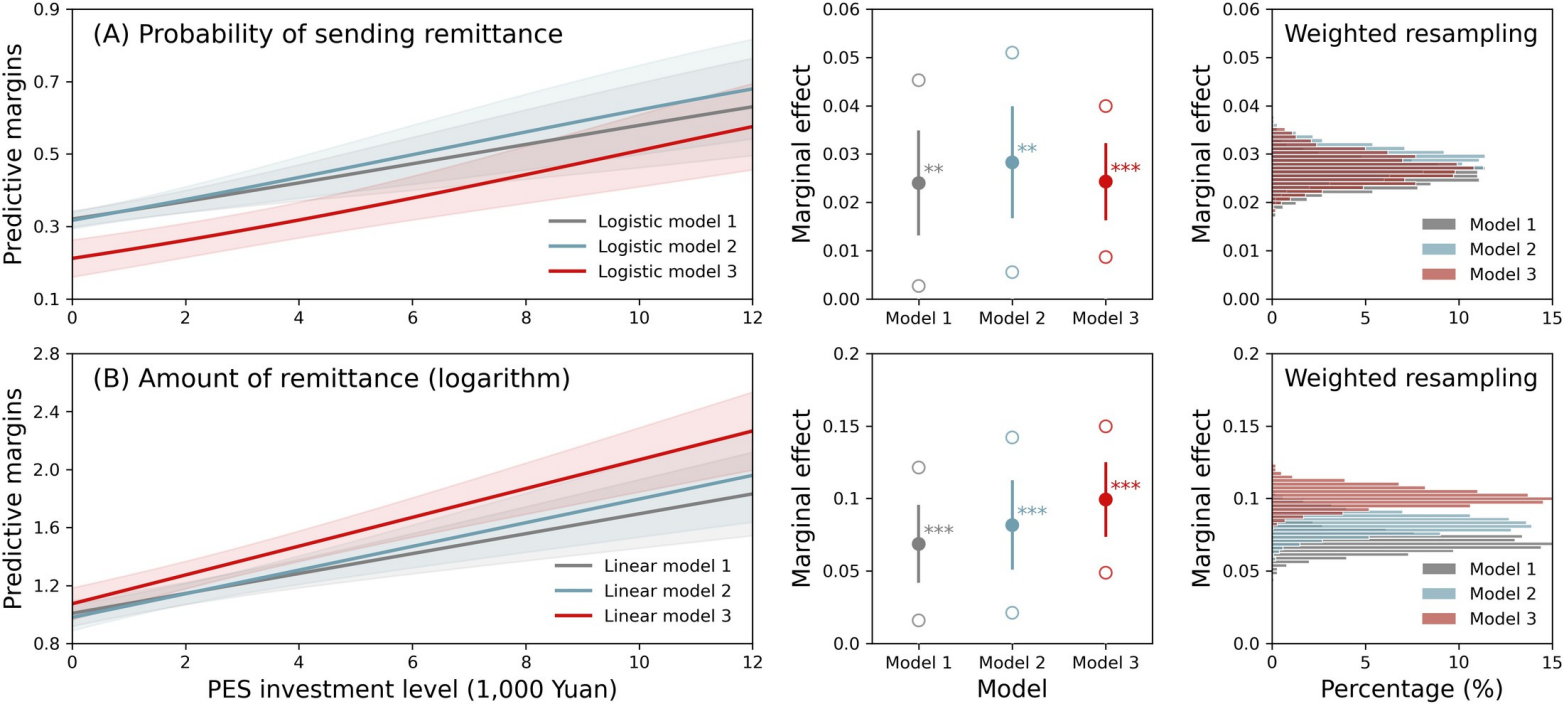
Effects of CCFP investments on sending remittance by individual migrants. Upper and lower panels are (A) probability of sending remittance and (B) remittance amount (logarithm, Yuan) based on multilevel analysis. Predictive margins (left panels) and marginal effects (middle panels) are estimated based on the individual sample; distributions of marginal effects (right panels) are estimated based on simulated population via weighted bootstrap sampling. For explanatory variables, Model 1 includes only CCFP; Model 2 adds individual attributes of migrants to Model-1; Model-3 adds characteristics of origin households to Model 2. 100 Yuan = US$16.08 (2014–2015). In the middle panels, ** *p* < 0.05; *** *p* < 0.01; bars represent standard errors; circles represent the 95% confidence intervals of the marginal effects of CCFP.

According to the multilevel mixed-effects models, CCFP payments have significant positive effects on the likelihood of sending remittance by and the amount of remittance from migrants to their origin households (**Fig 2**). The effects are robust when controlling for individual attributes, household characteristics, and community-level factors (**S2 and S3 Tables**). Setting the confounding factors at their means, every additional 1,000 Yuan (US$160.8) of CCFP payments have marginal effects of 0.024 and 0.099 on the probability of sending remittance and the remitted amount, respectively (Model 3, **S4 and S5 Tables**). With an increase in the accumulated investment level of CCFP from 0 to 12,000 Yuan (US$1,929.3) during the migration years, the probability of a migrant sending remittance increases from 0.21 to 0.58, and the mean remittance amount grows from 59.5 to 920.5 Yuan (or from US$10 to US$148) over a 5-year period of migration on average. Based on the weighted bootstrapping method (**S2 Text**), the estimated marginal effects are consistent across the models that include different sets of explanatory variables at the individual, household and community levels.

### 3.3. Effect of remittance on forest change surrounding origin households

**Fig 3:**
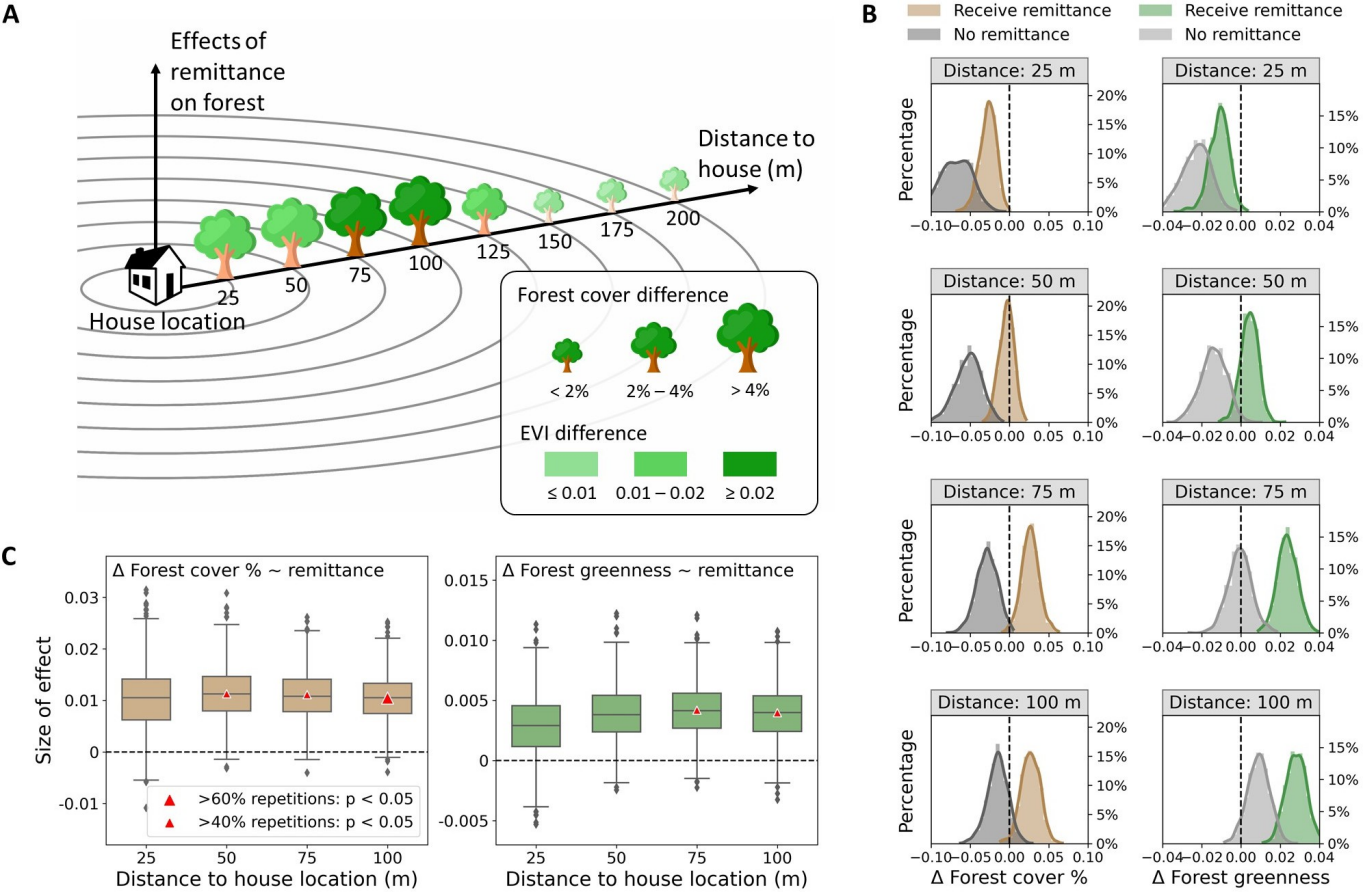
Effect of remittance by migrants on change in forest coverage and greenness. (A) Average treatment effects of remittance on forest change measured by the averaged differences of changes of forest coverage and greenness between the matched households receiving remittance and not receiving remittance within buffer circles from 25 m to 200 m. (B) Distributions of forest dynamics for households receiving remittance and those not, with comparisons of the forest indicator values for the two household groups to zero within buffer circles from 25 m to 100 m. (C) Effect sizes of remittance on forest dynamic within buffer circles from 25 m to 100 m.

By matching households receiving remittance with those having migrants but not receiving remittance with propensity scores, the estimated average treatment effects showed that forests surrounding households with remittance have experienced larger increases in forest cover and greenness than those surrounding houses receiving no remittance (**Figs 3A and S5**), during the study period 2002-2013/14. The effects are most prominent within the buffers of 75–100 m. Within the 100-m buffer surrounding the sampled households receiving remittance, for instance, the proportion of forest coverage is 4.2% ± 1.9% greater (>68% occurrence of *p* < 0.05) and the mean forest greenness measured by Enhanced Vegetation Index (EVI) is 0.02 ± 0.01 higher (>76% occurrence of *p* < 0.05) than those within the same buffer distance surrounding households with migrants but receiving no remittance. Within shorter buffer distances of 25–50 m, we also observed significantly higher increases of forest cover (>4%) and forest EVI (0.01∼0.02) (>65% occurrence of *p* < 0.05) for the household groups receiving remittance.

To separately assess the extent of forest change surrounding the migrant households with and without remittance, we plotted the distributions of changes in forest cover and mean forest EVI values from bootstrapping samples of 1,000 repetitions (**Fig 3B**). Significant differences exist between the two groups of households. In the immediate proximity of the house locations (25–50 m), we found no significant forest change for households receiving remittance but did find forest shrinkage and browning surrounding households without remittance (**Fig 3B**). Within the 75 m and 100 m buffer zones, forests around households with remittance showed significant changes, with forest area expansion at the 100 m buffer of 2.7% ± 1.2% (>60% occurrence of *p* < 0.05), and a greenness increase 0.03 ± 0.005 (>99% occurrence of *p* < 0.05). In contrast, within the same buffer ranges around households without remittance we found forest shrinkage of 1.5% ± 1.3% on average, albeit with low occurrence of *p* < 0.05 of only 17%.

Strong positive associations exist between the amount of remittance received and forest changes, particularly for the buffer ranges of 75 m and 100 m (**Fig 3C**). A 10-times increase in remittance received by the household is associated with an increase of 1.0% ± 0.4% in forest coverage proportion within the 100-m buffer circle, amounting to an area of 564.3∼1,403.5 m^2^ of forest. Such positive associations remain robust for the averaged amount of remittance by the number of migrants from the same household (**S5 Fig**).

In addition, we tested the sensitivity of the treatment effects with a range of caliper thresholds that define the closeness of the two matched households regarding their propensity score (**S2 Text**). The smaller the threshold, the fewer the matched observations available, as it is harder to find a match at a smaller caliper, leading to a relatively small number of samples retained for the statistical tests. We found that the treatment effects are rather stable across all caliper ranges when the buffer size is at least 75m, which is the range exhibiting the most prominent forest expansion and greening around the house locations (**S6 Fig**). These analyses support the three hypotheses regarding remittance effects on forest change within various ranges in proximity.

**Fig 4:**
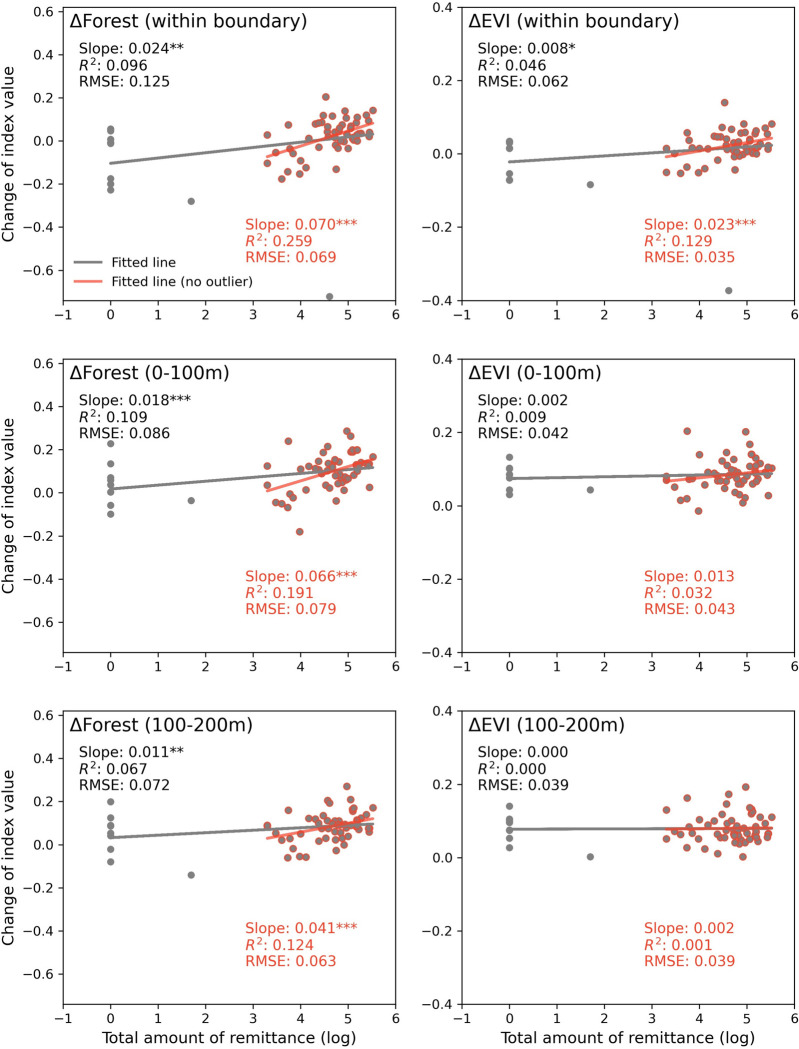
Scatter plots of total amount of remittance and forest change by different buffer ring distances at the group level. The gray trend line is estimated based on all group points while the red trend line on points excluding outliers. * *p* < 0.1; ** *p* < 0.05; *** *p* < 0.01.

Forests surrounding a household may also be influenced by the neighboring household’s use of forest resources [55]. To address the spatial overlap effects, we aggregate the remittance data to the resident group level where natural clusters of households situate (**S7 Fig**). There were a total of 63 spatial clusters representing household groups with 22 and 41 groups in J&C (Shanxi) and TTZ (Anhui), respectively. On average, the remittance received by each group in TTZ (104,516 Yuan or US$16,803) is much higher than that in J&C (14,138 Yuan or US$2,273). Scatterplots of remittance (log transformed) and forest change (cover and EVI) show that the association of forest change with remittance at the group level remains positive, particularly for forest-cover changes (**Fig 4**). For example, higher amounts of remittance are significantly correlated with both expanded and greener forests within the group boundaries; such relationships hold for forest-cover change when the buffer extends to 0-100m and 100-200m outside the boundaries of resident groups.

### 3.4. Mechanism analysis of remittance effects on forest dynamics

To explore the mechanisms by which remittance affects forest change, we compared the forest-dependent livelihood activities between the household groups with and without remittance (**Table 2**). We found that households receiving remittance used significantly less fuelwood in their total energy consumption (t = -8.25, *p* < 0.01), and more households among those receiving remittance adopted modern fuels (e.g., natural gas) than fuelwood (χ^2^ = 5.55, *p* < 0.10). The share of fuelwood consumption (approximated in estimated values) in total energy use, along with the share for daily cooking, was significantly lower for households receiving remittance or for those receiving more remittance. Moreover, in our northern study site (J&C), remittance was negatively associated with the number of domestic livestock kept by households (coefficient = -0.32, *p* < 0.1), while in the central-southern study site (TTZ), households receiving remittance had significantly less costs of inputs (e.g., hiring labor) for extracting forest resources (t = -7.35, *p* < 0.01). A huge amount of fuelwood is often used to conduct daily activities especially cooking and feeding livestock [39]. Remittance tends to expand fuel choices and reduce households’ reliance on forest resources.

**Table 2:** Mechanism tests for using forest resources in relation to remittance from migrants.

Variable	Comparison between households receiving remittance and households not receiving remittance from migrants				Relationship with remittance amount
	Obs. (1)	Obs. (0)	Expected	Test	
• Main energy source is fuelwood (and/or coal)	79.9%	80.3%	80.1%	χ^2^ = 5.55*	NA
• Half and half for fuelwood (and/or coal) and gas (e.g., natural gas, LPG)	5.9%	7.1%	6.6%		
• Main energy source is gas (e.g., natural gas and/or LPG)	14.2%	12.7%	13.3%		
	Mean (1)	Mean (0)	Difference	Test	Coef. (Std. Err.)
Share of fuelwood in energy cost	0.91	0.95	-0.04	t = -8.25***	-0.078 (0.041) ***
Share of fuelwood (cook) in energy cost	0.88	0.95	-0.06	t = -10.43***	-0.095 (0.047) ***
Whether raising livestock, J&C (0/1)	0.355	0.378	-0.023	t = -1.035	-0.319 (0.163) *
Whether raising livestock, TTZ (0/1)	0.800	0.791	0.009	t = 0.615	0.057 (0.073)
Cost of inputs for extracting forest resources, TTZ (1,000 Yuan)	1.172	2.927	-1.755	t = -7.349***	0.059 (0.271)

Notes:

* *p* < 0.10

** *p* < 0.05

**Fig 5:**
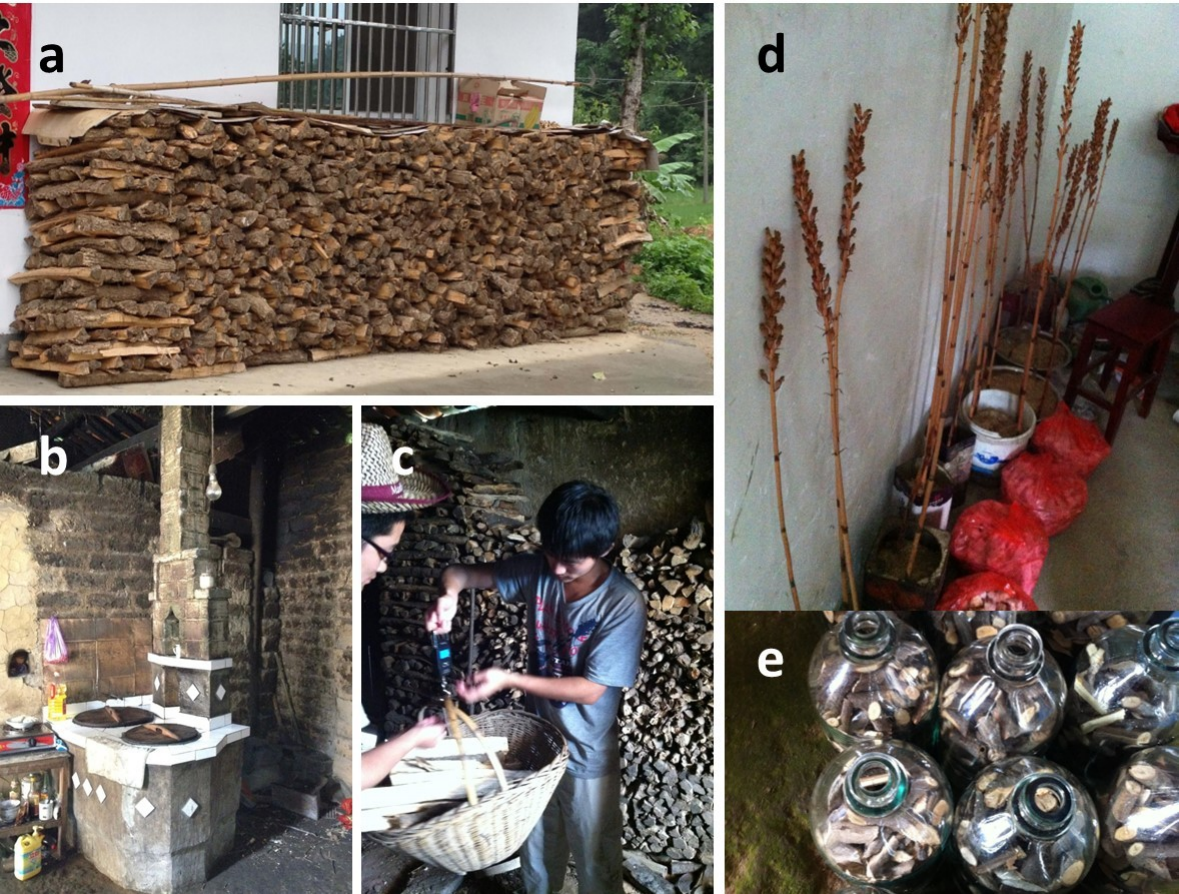
Fuelwood use as an energy source by rural households during the household survey. (a) Stack of fuelwood collected by a household from natural forest nearby. (b) Stove for cooking daily meals using fuelwood as the energy source. (c) Measuring weight of fuelwood used for cooking meals on an average day reported by a respondent in the survey. (d) *Gastrodia Elata* (GE), a major cash crop in TTZ (Anhui). (e) GE spores inoculated on freshly cut woods. Credit: First and corresponding authors.

*** *p* <0.01. The share of fuelwood in energy cost is estimated as the proportion of fuelwood usage in the summed use of fuelwood and gas (e.g., natural gas, liquefied petroleum gas). Score and stage of energy use are calculated following Song et al. [39]. Forest resources exclude fuelwood. Data for forest resources in J&C were not available (NA), while one major forest resource in TTZ is *Gastrodia Elata* which needs fuelwood for seed planting (**Fig 5**). Relationships are calculated based on ordinary least square regression models. Sample weights are applied.

### 3.5. Socioeconomic and ecological additionality of CCFP through remittance

**Fig 6:**
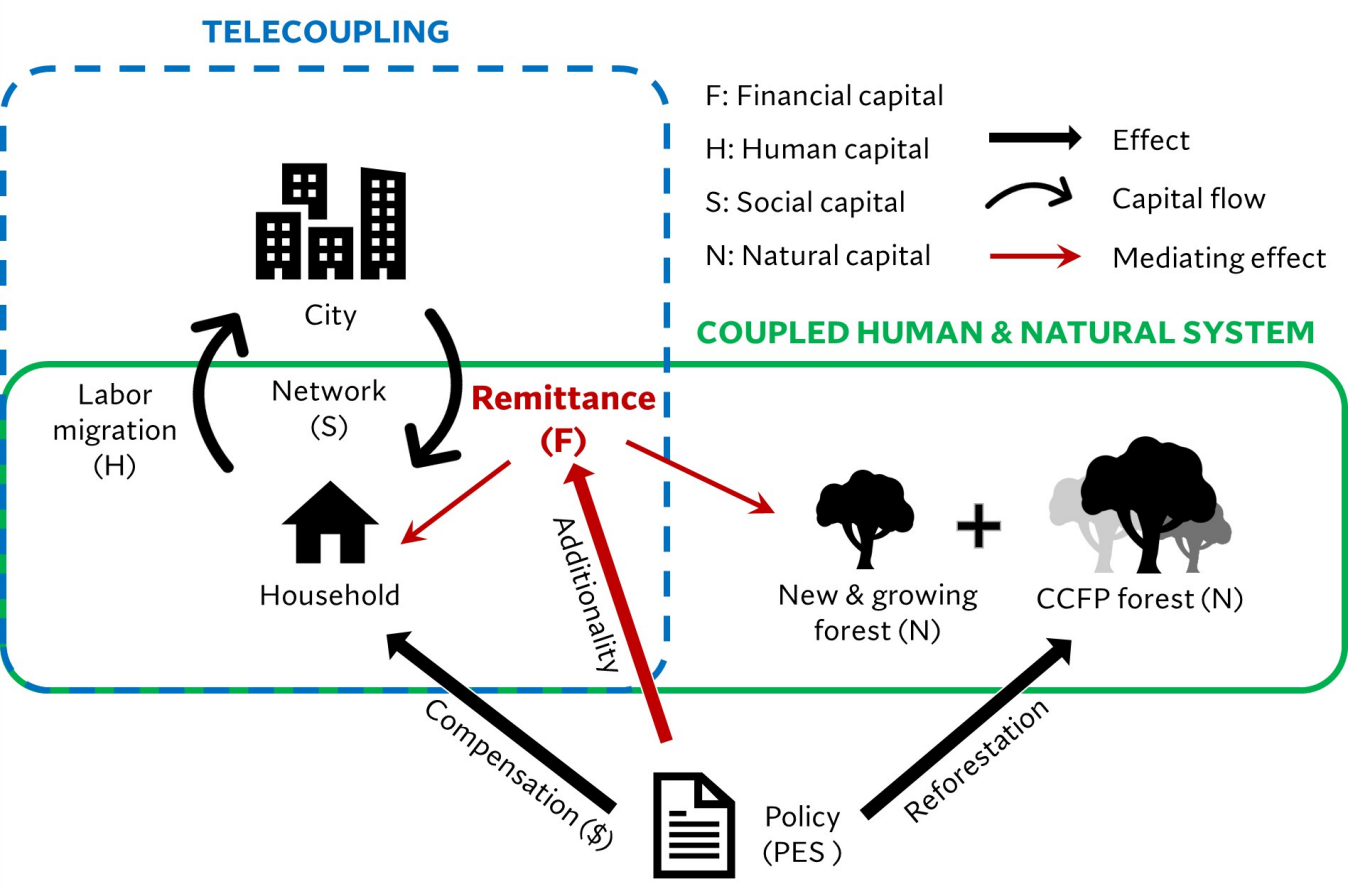
Synthesis of the mediating role of remittance in sustaining socioeconomic-ecological effects of PES investment on household livelihood and forest recovery. Telecoupling is characterized by the exchange of human capital (farm labor) and financial capital (remittance) between origin households and distant cities, and emerging social networks. The coupling of human and natural systems is featured by livelihood-forest dynamics with feedbacks. For instance, CCFP retires cropland for reforestation and hence frees farm labor from land cultivation to alternative livelihoods including labor migration, which can feedback to forest recovery mediated by remittance.

Finally, we estimated the socioeconomic and ecological additionality of the PES program that are mediated by remittance (**Fig 6**). The socioeconomic additionality is the proportion of CCFP-stimulated remittance over the total CCFP investment; the ecological additionality is the share of remittance-induced new forest area in addition to the baseline CCFP forest area. The socioeconomic and ecological additionalities of the CCFP investment mediated by remittance were estimated to be 2.0% (1.4%∼3.8%) and 9.7% (5.0%∼15.2%), respectively, based on our household samples. According to our estimates and the official reports by the National Forestry and Grassland Administration [25], the CCFP investment would stimulate nationwide an additional flow of US$0.9∼2.4 billion of remittance from migrants originated from households enrolled in the program and 1.5∼4.5 million ha of new forested land under the policy intervention; the estimated size of the remittance-mediated forest regeneration accounts for 12.7% (6.0%∼18.0%) of the total new forest gained during the 2003–2013 in China [56].

## 4. Discussion

Payment for environmental services (PES) has been practiced globally as an innovative way to achieve social-ecological sustainability. The world’s largest PES program, the Conversion of Cropland to Forest Program (CCFP) in China, is one of such efforts considered to have had more successes than setbacks [24,26,57,58]. With the goal of ecological restoration, the program engages rural households with far-reaching impacts on their livelihoods [59], including the rural out-migration strategies. Our results provide evidence that remittance by migrants can reinforce the socioeconomic and ecological gains of the CCFP, enhancing the sustainability of both forest recovery and rural livelihoods (**Fig 6**). Forest dependent households are the land stewards in PES programs [60]. Labor out-migration is a primary strategy for participating households to mitigate the income loss from withdrawing cropland from production. However, it comes with risks. Rural migrants in cities often face marginalization due to various obstacles such as health insecurity and mental stress [61,62]. Were the migrants not successful in pursuing employment that generates remittance, the origin households would rarely be able to shift or diversify their livelihoods relying on forests.

An interesting finding in this study is that migration without the mediator of remittance may not be effective in driving forest recovery or preventing deforestation. In the absence of remittance, the dual loss of human capital (farm labor) and natural capital (cropland) would make local livelihoods even more vulnerable, forcing the remaining household members to become more dependent on forest resources, such as using fuelwood as the major energy choice [39] as also observed in other developing countries [63]. Feedback loops built upon such adverse coupling of human and natural systems may produce unintended consequences, such as the “poverty trap”, whereby the rural poor are left with the increasingly degraded forests [64].

Our research conceptually advances knowledge of telecoupling and coupled human and natural (CNH) systems and have practical implications for forest conservation as a major strategy of climate change mitigation. Remittance serves as the medium that telecouples faraway cities and the migrant origin places (**Fig 1**), rendering the local CNH systems as both a sending (migrants) and receiving (remittance) system [8,32]. PES investments can stimulate additional remittance from migrants, which has social and environmental implications. The CCFP helps initiate or accelerate the process of rural-to-urban migration as the withdrawal of cropland leads to farm-labor surplus [65]. Meanwhile, CCFP compensation for cropland retirement acts as a financial insurance for the migrants to build social connections, develop skills, overcome barriers, and be relatively competent in non-farm job markets, particularly during the initial years in far-away cities [66]. Remittance can effectively break the dependence of the origin households on natural capital for livelihoods, and substantially contributes to forest regeneration [67]. Furthermore, areas surrounding remittance-receiving households experienced increased forest area and greenness due to their declined dependence on fuelwood and fodder, which suggest increase in carbon sink. Therefore, supporting remittance-sending migrants can be a major global warming mitigation strategy as rural-to-urban migration is a global phenomenon, not unique to China.

Forest conservation programs should not only invest their funds and efforts in ecosystem services, but also in people’s livelihoods, which can further strengthen ecosystem services through their reciprocal relationship with nature [68]. Although labor migration has been regarded as a primary driver of poverty alleviation and reduced deforestation [14], we highlight the mediator of remittance as a key glue in forest-livelihood dynamics of environmental, socioeconomic, and political relevance for various stakeholders. Migration without remittance does not break the original households away from reliance on natural resources for livelihoods. Thus, deforestation and forest degradation are likely to persist in places where households have sent migrants to distant cities yet not received remittance, as seen in this study. To address the grand challenge of such socio-environmental interactions in forest restoration and preservation, it is imperative to catalyze or facilitate a shift from environmental governance to governance for sustainability [69]. The forest conservation policy alone is short-handed. Companion policies that aim at improving the welfare of rural migrants in cities so that they can send remittance to the origin households are highly desirable for reinforcing socioeconomic and ecological gains. For example, education opportunities for migrants to acquire new employment skills and for their children, and affordable health care are among the low-hanging fruits to help migrants to succeed and send back remittance. Achieving sustainable forest restoration requires a constellation of coordinated policies that comprehensively address issues from the social and environmental systems that are ever-increasingly integrated across multiple scales through time.

We acknowledge that there exist limitations in the current study. Although our models control for the confounding effect of migration years, the primary information on remittance is based on cross-sectional data obtained from the household survey in a given year. Our analysis thus does not track the behavioral dynamics of sending remittance by out-migrants. Testing this time-varying variable would require not only the longitudinal data on remittances received by the rural households at the migration origin, but also the detailed information on migrant activities in cities (e.g., promotion, job change) from the destination migrant destination location. Given our focus on forest change and rural livelihoods for origin households, extending the data collection in these two domains would be beyond the scope of this study and can be conducted in future work. Another limitation is that this study does not consider alternative forms of migration, such as seasonal migrants, return-migrants, and the moving of out-migrants between cities without returning home [70,71]. We define out-migrants as those living and working outside the county boundary for more than six consecutive months and remaining away from home at the time of interview, indicating their absence when the origin households conducted livelihood activities such as land cultivation. However, seasonal migrants who live closer to the study site (yet still outside the county boundary), for instance, may frequently return home to help with forest livelihoods (e.g., fuelwood collection), potentially incurring bias for the estimated effect of the remittance on forest change. Future studies can test these effects by categorizing out-migrants into different types relating to their contribution to their origin households.

## 5. Conclusions

This study evaluates the mediating effect of remittance on forest restoration. We find significant forest-cover increase and greening surrounding migrant households receiving remittance, but forest-cover decrease and browning surrounding migrant households not receiving remittance. The effect of the remittance on forest restoration can also be detected at the resident group level. Households enrolled in China’s Conversion of Cropland to Forest Program (CCFP) are more likely to receive remittance, generating additional benefits in both the social and environmental systems. Through the mediation of remittance, CCFP generates additional 2.0% (1.4∼3.8%) income to the enrolling households and 9.7% (5.0–15.2%) of new forests through natural regeneration above the CCFP baseline forest area. Scaling up such effects to the national level, China’s CCFP is estimated to stimulate an additional US$0.9–2.4 billion of remittance from migrants and additional 1.5∼4.5 million ha of new forests, or 12.7% (6.0∼18.0%) of the total new forests established during 2003–2013. The key mechanism of remittance mediation is the reduction of reliance on natural resources for rural livelihoods, such as fuelwood or fodder. We argue migrants and remittance are the critical telecoupling linkages between the migrant origin places and the migrant destination cities. A significant policy implication from this study is that implementing forest restoration or conservation policy alone may not generate sustainable ecosystem services (e.g., carbon sequestration by healthy forests), and companion socio-economic policies that support the livelihoods of migrants in the cities can improve the environment through telecoupling. The additional ecological and socioeconomic gains brought by remittance to the forest policy help achieve sustainability of the coupled human and natural system, simultaneously facilitating forest regeneration and improving local livelihoods. Given that rural out-migration is a global phenomenon, the world will reap significant environmental benefits by supporting rural migrant’s successes, e.g., technical training and health care, in cities around the globe.

## Supporting information

S1 Fig Study sites of Jichangzhen & Chechengxiang (J&C) and Tiantangzhai (TTZ) and the spatial distribution of the surveyed households. The J&C site (the upper right panel) is located in Shanxi Province of in northern China on the Loess Plateau with a semi-arid climate. The TTZ site (the lower right panel) is located at the Dabieshan mountain ranges in western Anhui of central-eastern China with a subtropical monsoon climate. Elevation maps are generated using the Shuttle Radar Topography Mission digital elevation data.
(PDF)

S2 Fig Migration and remittance flows tracked between study sites and distal regions over the country with migration destinations set at the provincial capital cities. JC and TTZ represent two study sites. A wider arrow indicates a larger volume in migration (number of migrants) or remittance (amount). A higher opacity level of an arrow indicates a longer distance. The ranges of distance (logarithm) are 2.1∼2.6 (short), 2.6∼2.9 (medium), 2.9∼3.2 (long) at the provincial level. ANOVA tests were used to test difference among short, medium and long distant migration (Number of migrants: F = 10.16, p < 0.01; Amount of remittance: F = 2.00, p = 0.16; Remittance per migrant: F = 1.24, p < 0.31. Data source for forest changes during 2000–2015 is Global Forest Cover Change by Sexton et al. (2013) [53]. A forest pixel is defined as that the fraction of forest cover is greater or equal to 30% following the criteria by Hansen et al. (2013).
(PDF)

S3 Fig Pearson Correlation between explanatory variables at multiple levels. (PDF)

S4 Fig Histograms of (a) estimated propensity scores for the two household groups with (treated) and without (control) remittances before matching (upper panel), and (b) score difference after matching (lower panel). In the upper panel, accuracy is calculated as the percentage of the number of correctly predicted scores (0.5 or above for sending remittance and below 0.5 for not sending remittance) to the total number of scores. In the lower panel, t-test shows that the mean value of score difference of the matched households does not significantly deviate from zero (t = -0.42, p = 0.68).
(PDF)

S5 Fig Effects of remittance on forest coverage and greenness change. Panels (a) and (b) are differences in temporal change of forest cover proportion and mean forest EVI, respectively, between matched households with and without remittance by various sizes of buffer zones around the household residence. Panels (c) and (d) are associations of forest changes with the total remittance amount and the averaged remittance from migrants, respectively.
(PDF)

S6 Fig Differences of forest cover and greenness changes in the buffer around households with and without remittance by various levels of caliper for matching. To match each household in the control group, a treated counterpart is randomly selected from households that possess scores within the caliper threshold of the score of the control household.
(PDF)

S7 Fig Spatial maps of forest cover change with matched household locations and the resident group boundaries in J&C and TTZ. In J&C, one household and two group boundaries are outside the study site boundary, thus forest cover changes within the 1-km buffer are included for the analysis. Forest change maps are generated using Landsat OLI and ETM+ satellite images.
(PDF)

S1 Table Descriptions of explanatory variables for modeling remittances sent by out-migrants. Notes: The area unit, mu, measures the size of cropland in China; 1 mu = 666.7 m2. US$1 ≈ 6.22 Yuan (2014–2015). Scores of house condition, farm tools, and transportation evaluate the physical capital endowment of a household reflecting the wellness status (Song et al. 2018). SD denotes standard deviation. TTC represents the Tiantangzhai site and J&C represents the Jichang and Checheng site. The selection of multilevel factors follows both theoretical and empirical understanding of labor migration and household livelihood (Carney et al. 1999 [41]; Ostrom 2009 [35]), which have been summarized in (Zhang et al. 2018 [44]).
(PDF)

S2 Table Estimation of multilevel mixed-effects logistic model on whether out-migrants sending remittances. Notes: * p<0.1; ** p<0.05; *** p<0.01. Sample weights are applied in the regression. Values in parentheses are standard errors. ICC represents the intraclass correlation, calculated as *Σ*/(*γ*+*Σ*), where *Σ* is the random intercept variance and *γ* is the residual variance (i.e., *π****2***/3). AIC and BIC represent Akaike’s and Schwarz’s Bayesian information criteria, respectively. A level of difference greater than 10 in BIC or AIC values between two models suggest that the model with the lower value is favored (Claeskens & Hjort, 2008). Model 3 with the lowest AIC and BIC values is selected for further analysis.
(PDF)

S3 Table Estimation of multilevel mixed-effects model on the amount of remittances sent by out-migrants. Notes: * p<0.1; ** p<0.05; *** p<0.01. Sample weights are applied in the regression. Values in parentheses are standard errors. ICC represents intraclass correlation, calculated as *σ****τ***2/(*γ*+*σ****τ***2) where *σ****τ***2 is the random intercept variance and *γ* is the residual variance. AIC and BIC represent Akaike’s and Schwarz’s Bayesian information criteria, respectively. A level of difference greater than 10 in BIC or AIC values between two models suggest that the model with the lower value is favored (Claeskens & Hjort, 2008). Model 3 with the lowest AIC and BIC values is selected for further analysis.
(PDF)

S4 Table Marginal effects of explanatory variables for multilevel mixed-effects logistic modeling on whether out- migrants sending remittances based on Model-3 (Explanatory variables: CCFP + individual attributes + household characteristics). (PDF)

S5 Table Marginal effects of explanatory variables for multilevel mixed-effects linear modeling on amounts of remittances sent by out-migrants based on Model-3 (Explanatory variables: CCFP + individual attributes + household characteristics). (PDF)

S6 Table Selection of variables for matching households receiving remittances with households not receiving remittances. Notes: mu measures the unit management size of land in China; 1 mu = 666.7 m2. Scores of house condition, farm tools and transportation evaluate the physical capital endowment of a household reflecting the wellness status (Song et al. 2018) [39]. Values are the absolute proportion differences for binary variables and absolute standard mean differences for continuous variables. TTC represents the Tiantangzhai site and J&C represents the Jichang and Checheng site.
(PDF)

S1 Data (XLSX)

S1 Text (PDF)

S2 Text (PDF)
