# Supplementary material for: Remittance from migrants reinforces forest recovery for China’s reforestation policy
Source: PLoS One. 2024 Jun 26;19(6):e0296751. doi: 10.1371/journal.pone.0296751 (PMC11207146; doi:10.1371/journal.pone.0296751)
Supplement: S1 Fig — The J&C site (the upper right panel) is located in Shanxi Province of in northern China on the Loess Plateau with a semi-arid climate. The TTZ site (the lower right panel) is located at the Dabieshan mountain ranges in western Anhui of central-eastern China with a subtropical monsoon climate. Elevation maps are generated using the Shuttle Radar Topography Mission digital elevation data. (PDF) [file pone.0296751.s001.pdf]

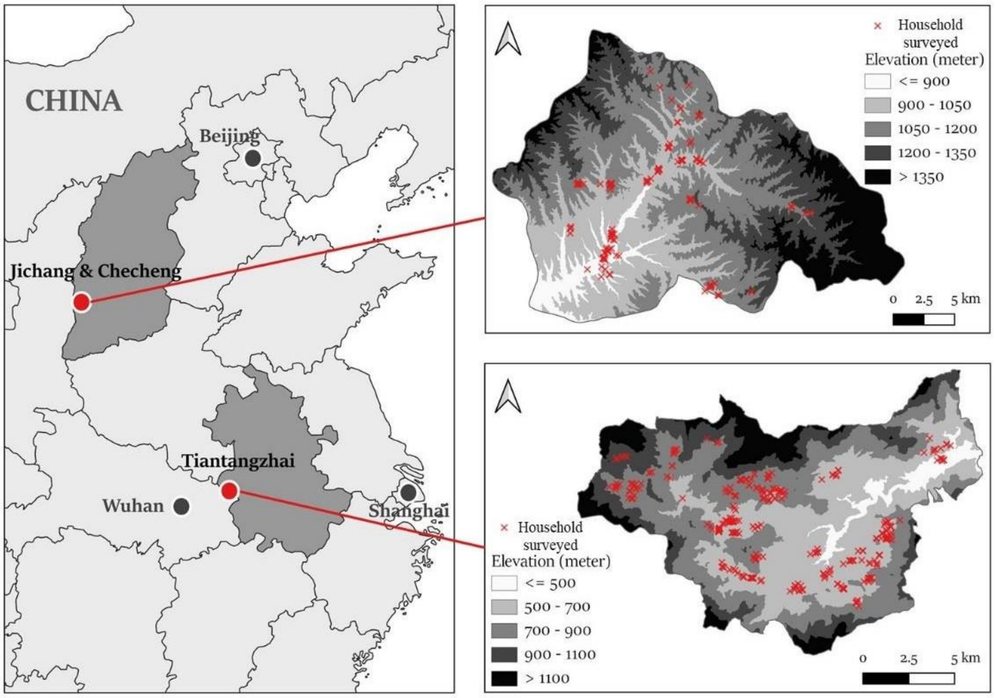


**Fig. S1.** Study sites of Jichangzhen & Chechengxiang (J&C) and Tiantangzhai (TTZ) and the spatial distribution of the surveyed households. The J&C site (the upper right panel) is located in Shanxi Province of in northern China on the Loess Plateau with a semi-arid climate. The TTZ site (the lower right panel) is located at the Dabieshan mountain ranges in western Anhui of central-eastern China with a subtropical monsoon climate. Elevation maps are generated using the Shuttle Radar Topography Mission digital elevation data.
