# Supplementary material for: Remittance from migrants reinforces forest recovery for China’s reforestation policy
Source: PLoS One. 2024 Jun 26;19(6):e0296751. doi: 10.1371/journal.pone.0296751 (PMC11207146; doi:10.1371/journal.pone.0296751)
Supplement: S2 Fig — JC and TTZ represent two study sites. A wider arrow indicates a larger volume in migration (number of migrants) or remittance (amount). A higher opacity level of an arrow indicates a longer distance. The ranges of distance (logarithm) are 2.1∼2.6 (short), 2.6∼2.9 (medium), 2.9∼3.2 (long) at the provincial level. ANOVA tests were used to test difference among short, medium and long distant migration (Number of migrants: F = 10.16, p < 0.01; Amount of remittance: F = 2.00, p = 0.16; Remittance per migrant: F = 1.24, p < 0.31. Data source for forest changes during 2000–2015 is Global Forest Cover Change by Sexton et al. (2013) [53]. A forest pixel is defined as that the fraction of forest cover is greater or equal to 30% following the criteria by Hansen et al. (2013). (PDF) [file pone.0296751.s002.pdf]

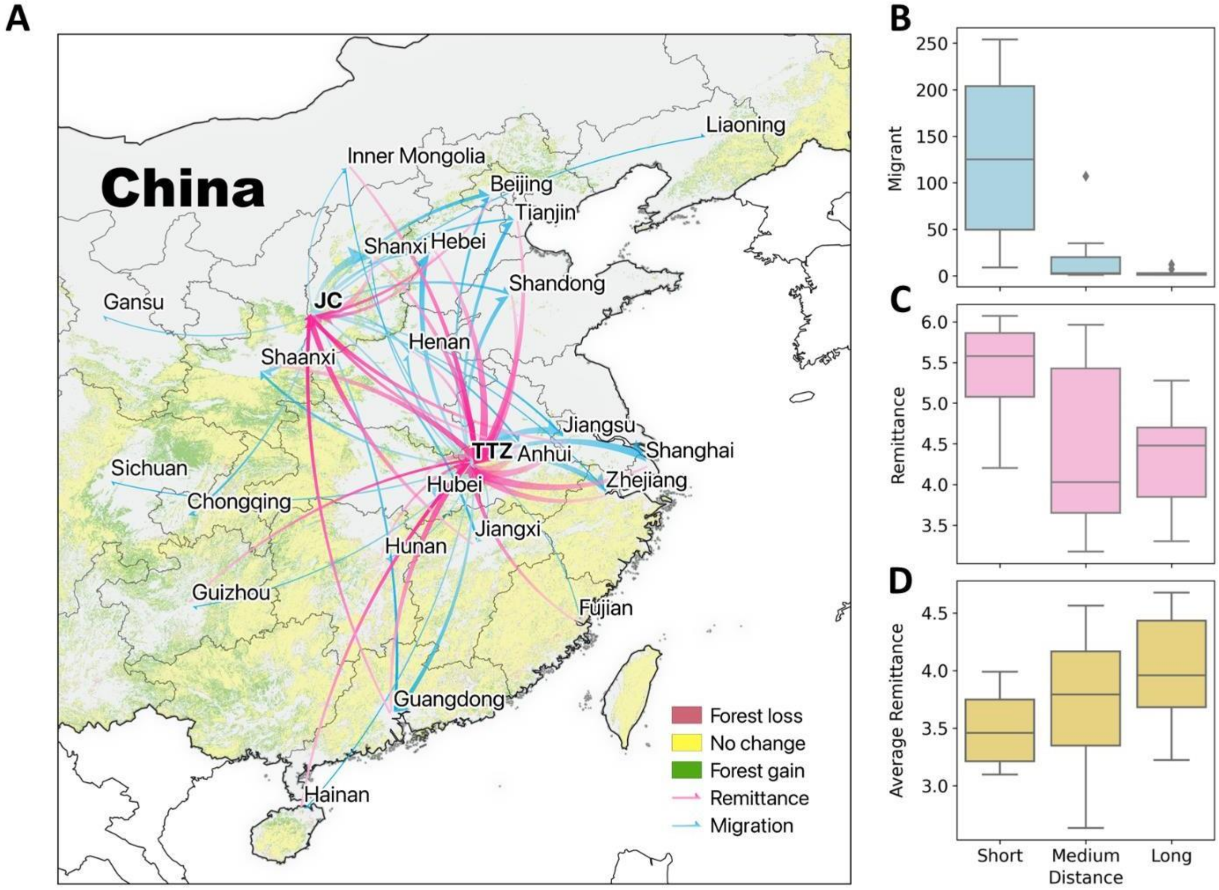

Fig. S2. Migration and remittance flows tracked between study sites and distal regions over the country with migration destinations set at the provincial capital cities. JC and TTZ represent two study sites. A wider arrow indicates a larger volume in migration (number of migrants) or remittance (amount). A higher opacity level of an arrow indicates a longer distance. The ranges of distance (logarithm) are 2.1~2.6 (short), 2.6~2.9 (medium), 2.9~3.2 (long) at the provincial level. ANOVA tests were used to test difference among short, medium and long distant migration (Number of migrants: $F = 10.16$, $p < 0.01$; Amount of remittance: $F = 2.00$, $p = 0.16$; Remittance per migrant: $F = 1.24$, $p < 0.31$. Data source for forest changes during 2000-2015 is Global Forest Cover Change by Sexton et al. (2013). A forest pixel is defined as that the fraction of forest cover is greater or equal to 30% following the criteria by Hansen et al. (2013).

References:

Sexton JO, Song X-P, Feng M, Noojipady P, Anand A, Huang C, et al. Global, 30-m resolution continuous fields of tree cover: Landsat-based rescaling of MODIS vegetation continuous fields with lidar-based estimates of error. International Journal of Digital Earth. 2013;6: 427–448. doi:10.1080/17538947.2013.786146

Hansen MC, Potapov PV, Moore R, Hancher M, Turubanova SA, Tyukavina A, et al. High- Resolution Global Maps of 21st-Century Forest Cover Change. Science. 2013;342: 850– 853. doi:10.1126/science.1244693
