# Supplementary figures and images for: Remittance from migrants reinforces forest recovery for China’s reforestation policy
Source: PLoS One. 2024 Jun 26;19(6):e0296751. doi: 10.1371/journal.pone.0296751 (PMC11207146; doi:10.1371/journal.pone.0296751)

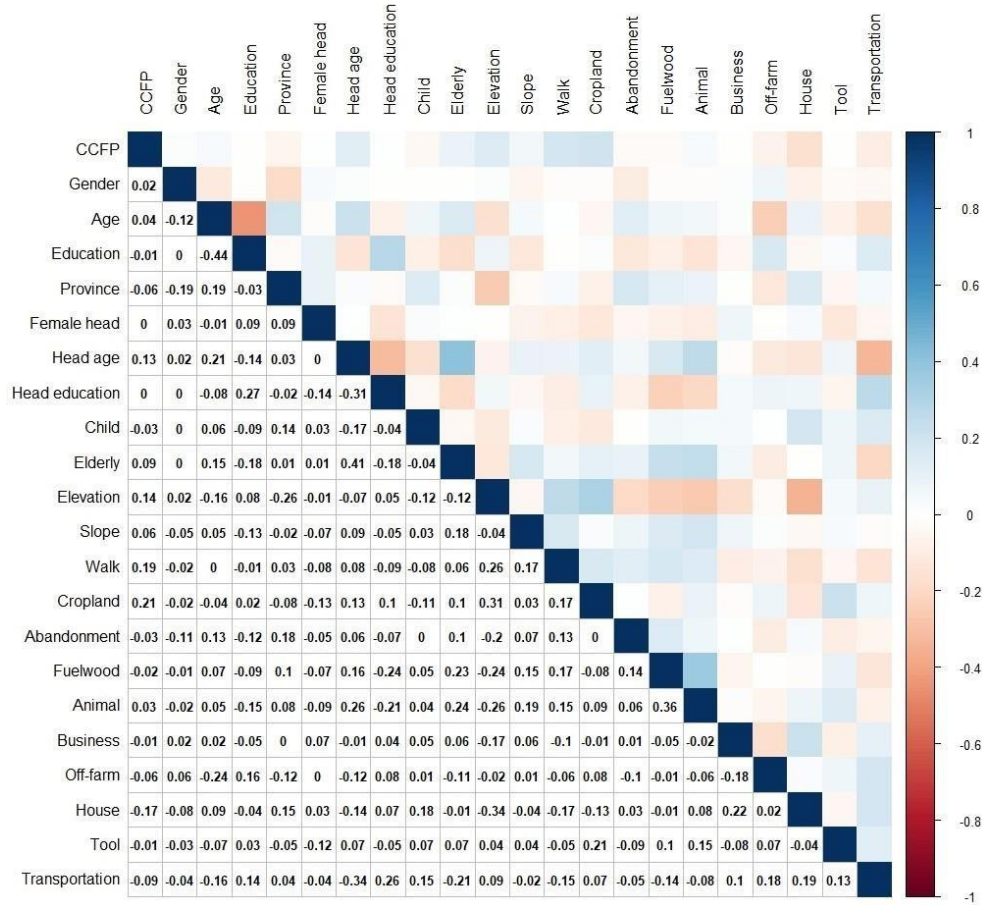

**Fig. S3.** Pearson Correlation between explanatory variables at multiple levels.

Supplement: S3 Fig — (PDF) [file pone.0296751.s003.pdf]
