# Supplementary material for: Remittance from migrants reinforces forest recovery for China’s reforestation policy
Source: PLoS One. 2024 Jun 26;19(6):e0296751. doi: 10.1371/journal.pone.0296751 (PMC11207146; doi:10.1371/journal.pone.0296751)
Supplement: S4 Fig — Histograms of (a) estimated propensity scores for the two household groups with (treated) and without (control) remittances before matching (upper panel), and (b) score difference after matching (lower panel). In the upper panel, accuracy is calculated as the percentage of the number of correctly predicted scores (0.5 or above for sending remittance and below 0.5 for not sending remittance) to the total number of scores. In the lower panel, t-test shows that the mean value of score difference of the matched households does not significantly deviate from zero (t = -0.42, p = 0.68). (PDF) [file pone.0296751.s004.pdf]

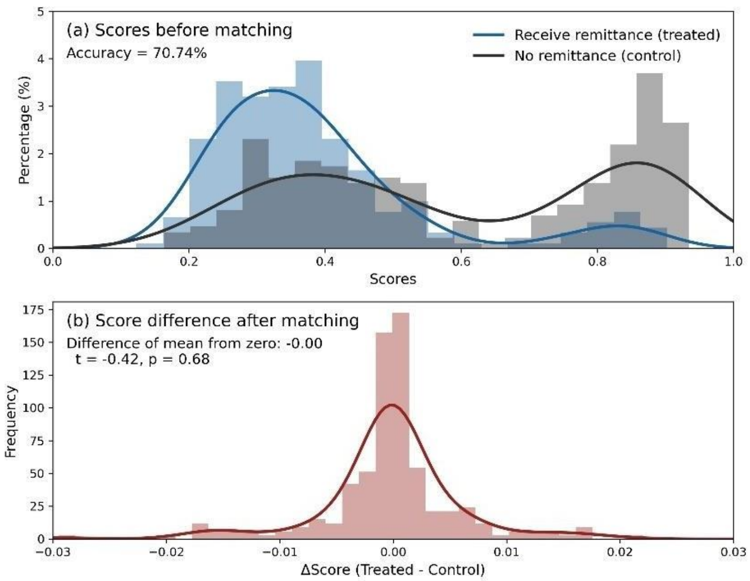

Fig. S4. Histograms of (a) estimated propensity scores for the two household groups with (treated) and without (control) remittances before matching (upper panel), and (b) score difference after matching (lower panel). In the upper panel, accuracy is calculated as the percentage of the number of correctly predicted scores (0.5 or above for sending remittance and below 0.5 for not sending remittance) to the total number of scores. In the lower panel, t-test shows that the mean value of score difference of the matched households does not significantly deviate from zero ($t = -0.42$, $p = 0.68$).
