# Supplementary material for: Remittance from migrants reinforces forest recovery for China’s reforestation policy
Source: PLoS One. 2024 Jun 26;19(6):e0296751. doi: 10.1371/journal.pone.0296751 (PMC11207146; doi:10.1371/journal.pone.0296751)
Supplement: S5 Fig — Panels (a) and (b) are differences in temporal change of forest cover proportion and mean forest EVI, respectively, between matched households with and without remittance by various sizes of buffer zones around the household residence. Panels (c) and (d) are associations of forest changes with the total remittance amount and the averaged remittance from migrants, respectively. (PDF) [file pone.0296751.s005.pdf]

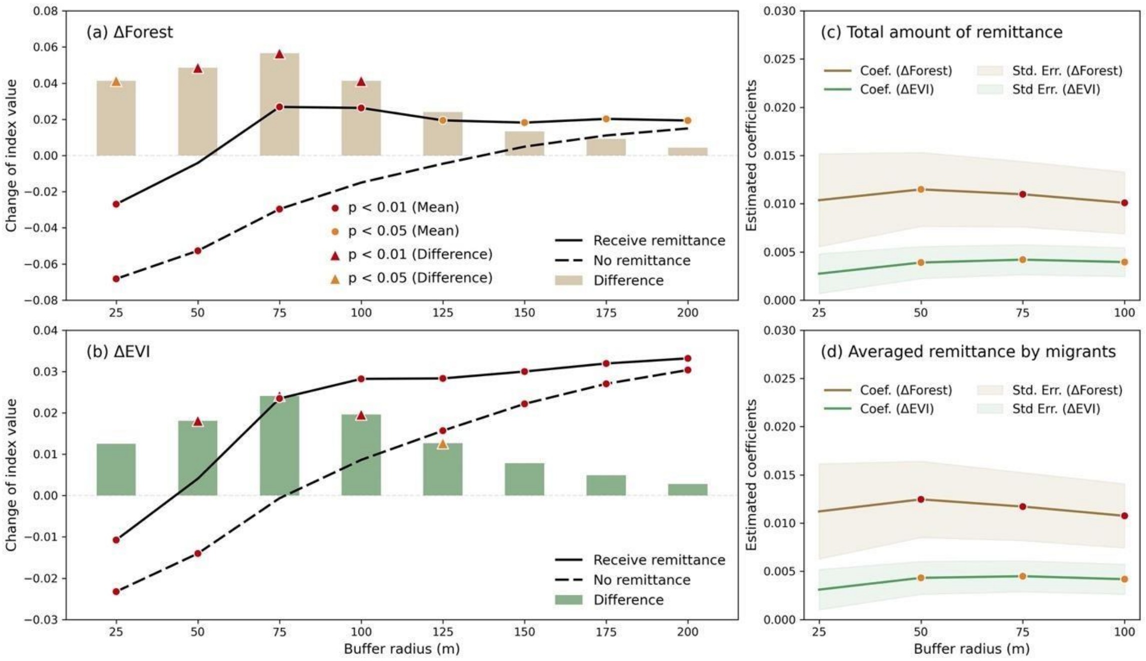


**Fig. S5.** Effects of remittance on forest coverage and greenness change. Panels (a) and (b) are differences in temporal change of forest cover proportion and mean forest EVI, respectively, between matched households with and without remittance by various sizes of buffer zones around the household residence. Panels (c) and (d) are associations of forest changes with the total remittance amount and the averaged remittance from migrants, respectively.
