# Supplementary material for: Remittance from migrants reinforces forest recovery for China’s reforestation policy
Source: PLoS One. 2024 Jun 26;19(6):e0296751. doi: 10.1371/journal.pone.0296751 (PMC11207146; doi:10.1371/journal.pone.0296751)
Supplement: S6 Fig — To match each household in the control group, a treated counterpart is randomly selected from households that possess scores within the caliper threshold of the score of the control household. (PDF) [file pone.0296751.s006.pdf]

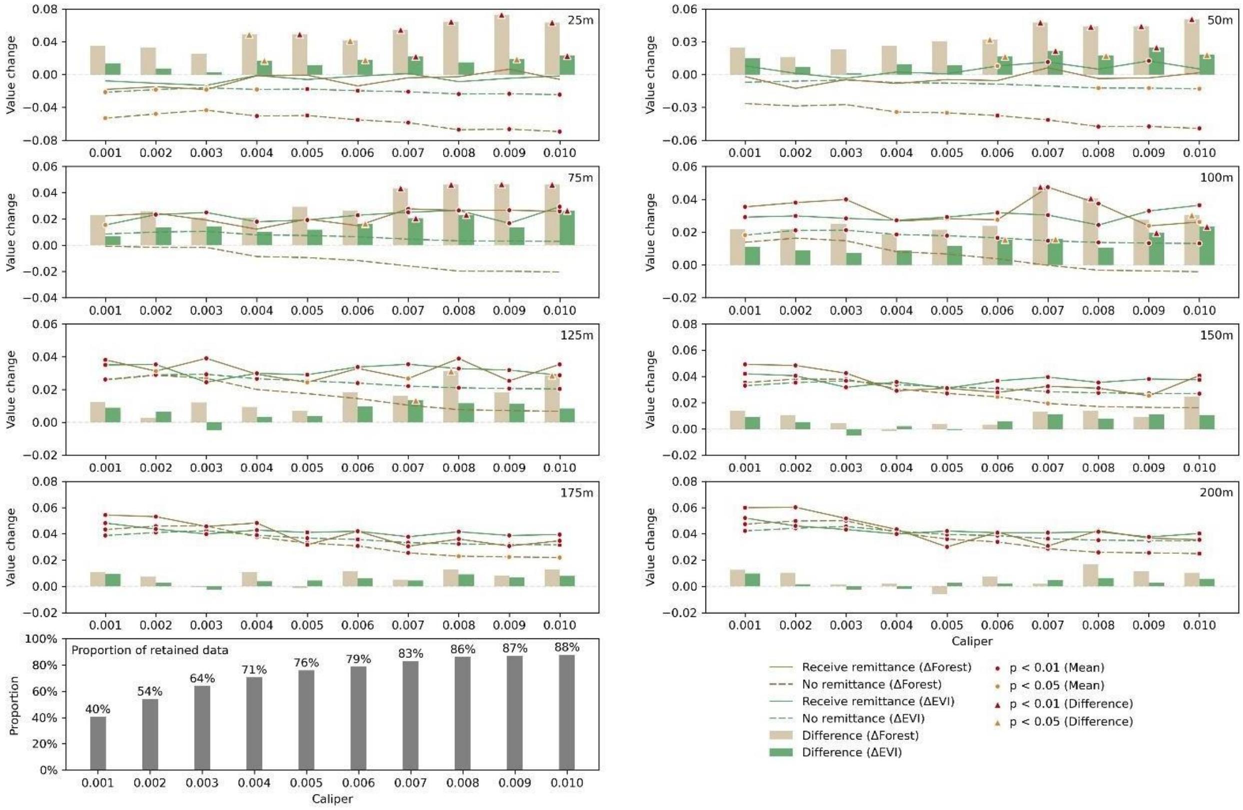

Fig. S6. Differences of forest cover and greenness changes in the buffer around households with and without remittance by various levels of caliper for matching. To match each household in the control group, a treated counterpart is randomly selected from households that possess scores within the caliper threshold of the score of the control household.
