# Supplementary material for: Remittance from migrants reinforces forest recovery for China’s reforestation policy
Source: PLoS One. 2024 Jun 26;19(6):e0296751. doi: 10.1371/journal.pone.0296751 (PMC11207146; doi:10.1371/journal.pone.0296751)
Supplement: S7 Fig — In J&C, one household and two group boundaries are outside the study site boundary, thus forest cover changes within the 1-km buffer are included for the analysis. Forest change maps are generated using Landsat OLI and ETM+ satellite images. (PDF) [file pone.0296751.s007.pdf]

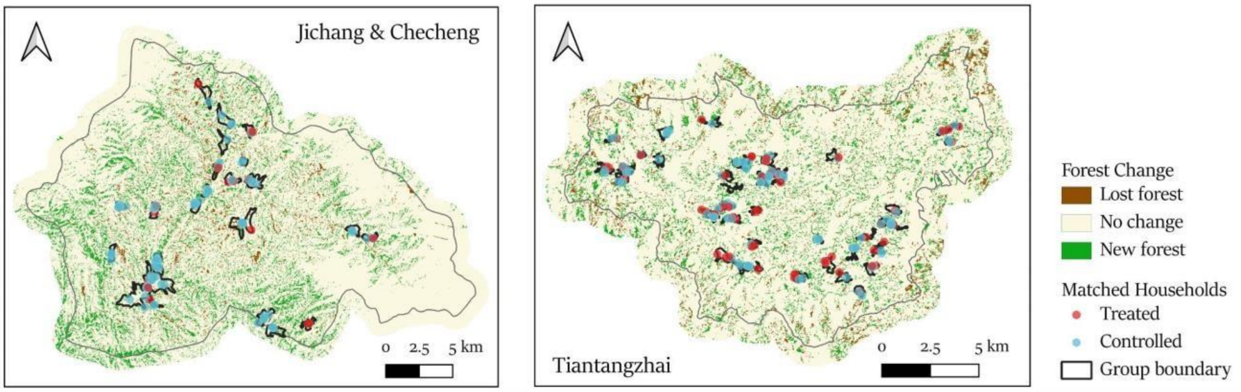

Fig. S7. Spatial maps of forest cover change with matched household locations and the resident group boundaries in J&C and TTZ. In J&C, one household and two group boundaries are outside the study site boundary, thus forest cover changes within the 1-km buffer are included for the analysis. Forest change maps are generated using Landsat OLI and ETM+ satellite images.
