# Supplementary material for: Remittance from migrants reinforces forest recovery for China’s reforestation policy
Source: PLoS One. 2024 Jun 26;19(6):e0296751. doi: 10.1371/journal.pone.0296751 (PMC11207146; doi:10.1371/journal.pone.0296751)
Supplement: S1 Table — Notes: The area unit, mu, measures the size of cropland in China; 1 mu = 666.7 m2. US$1 ≈ 6.22 Yuan (2014–2015). Scores of house condition, farm tools, and transportation evaluate the physical capital endowment of a household reflecting the wellness status (Song et al. 2018). SD denotes standard deviation. TTC represents the Tiantangzhai site and J&C represents the Jichang and Checheng site. The selection of multilevel factors follows both theoretical and empirical understanding of labor migration and household livelihood (Carney et al. 1999 [41]; Ostrom 2009 [35]), which have been summarized in (Zhang et al. 2018 [44]). (PDF) [file pone.0296751.s008.pdf]

Table S1. Descriptions of explanatory variables for modeling remittances sent by out-migrants.

Notes: The area unit, mu, measures the size of cropland in China; 1 mu = 666.7 m^2. US$1 ≈ 6.22 Yuan (2014-2015). Scores of house condition, farm tools, and transportation evaluate the physical capital endowment of a household reflecting the wellness status (Song et al. 2018). SD denotes standard deviation. TTC represents the Tiantangzhai site and J&C represents the Jichang and Checheng site. The selection of multilevel factors follows both theoretical and empirical understanding of labor migration and household livelihood (Carney et al. 1999; Ostrom 2009), which have been summarized in (Zhang et al. 2018; Zhang et al.2019).

**Variable**	**Description**	**Mean (SD)**
CCFP	Cumulative amount of compensation from CCFP with inflation adjusted to household during migration years for an individual migrant (100 Yuan)	1.37 (2.99)
Gender	Individual gender (1=female, 0=male)	0.47 (0.50)
Age	Age of individual (years)	29.59 (9.26)
Education	Education years completed in school	9.50 (3.58)
Province	Migration destination (1=outside origin province, 0=within origin province)	0.43 (0.50)
Female head	Gender of household head (1=female, 0=male)	0.07 (0.25)
Head age	Age of household head (years)	52.01 (9.49)
Head education	Education years household head completed	6.55 (3.04)
Child	Whether any household member is a child (age < 15) (1=yes, 0=no)	0.30 (0.46)
Elderly	Whether any household member is elderly (age > 59) (1=yes, 0=no)	0.41 (0.49)
Elevation	Elevation at house location (100m)	7.98 (1.87)
Slope	Slope at house location (degrees)	12.92 (6.92)
Walk	Walking distance from house to nearest paved road measured in time (minutes)	10.96 (15.86)
Cropland	Total area of cropland managed by household (mu)	6.59 (4.98)
Abandonment	Area of cropland the household abandoned (mu)	0.73 (1.37)
Fuelwood	Amount of fuelwood household used per year by household (1,000kg)	8.12 (6.67)
Animal	Whether raising domestic animals (1=yes, 0=no)	0.70 (0.460)
Business	Whether conducting local businesses (1=yes, 0=no)	0.08 (0.28)
Off-farm	Whether engaging off-farm activities within county (1=yes, 0=no)	0.52 (0.50)
House	Score of house condition (0-5)	2.62 (1.63)
Tool	Score of farm tools (0-5)	2.36 (1.68)
Transportation	Score of transportation tools (0-5)	2.54 (1.33)
Group size	Total number of households within a resident group	41.26 (32.64)
College	Number of individuals with college degrees within resident group	4.24 (6.27)
Hospital	Walking distance from resident group to nearest hospital or clinic (minutes)	17.04 (14.37)
School	Walking distance from resident group to nearest elementary school (minutes)	19.99 (21.26)
Study site	Study site (0=TTC, 1=J&C)	0.29 (0.46)
Migration years	Number of years from migration to survey year (2014 for TTC, 2015 for J&C)	5.25 (4.20)

References:

Hull V, Liu J. Telecoupling: A new frontier for global sustainability. E&S. 2018;23: art41. doi:10.5751/ES-10494-230441

Carney D, Drinkwater M, Rusinow T, Neefjes K, Wanmali S, Singh N. Livelihoods approaches compared: A brief comparison of the livelihoods approaches of the UK Department for International Development (DFID), CARE, Oxfam and the United Nations Development Programme (UNDP). London: Department for International Development; 1999 p. 19. Available: https://books.google.com/books/about/Livelihoods_Approaches_Compared.html?id=on4J MwEACAAJ

Ostrom E. A General Framework for Analyzing Sustainability of Social-Ecological Systems. Science. 2009;325: 419–422. doi:10.1126/science.1172133

Zhang Q, Bilsborrow RE, Song C, Tao S, Huang Q. Rural household income distribution and inequality in China: Effects of payments for ecosystem services policies and other factors. Ecological Economics. 2019;160: 114–127. doi:10.1016/j.ecolecon.2019.02.019

Zhang Q, Bilsborrow RE, Song C, Tao S, Huang Q. Determinants of out-migration in rural China: effects of payments for ecosystem services. Popul Environ. 2018;40: 182–203. doi:10.1007/s11111-018-0307-5
