# Supplementary material for: Remittance from migrants reinforces forest recovery for China’s reforestation policy
Source: PLoS One. 2024 Jun 26;19(6):e0296751. doi: 10.1371/journal.pone.0296751 (PMC11207146; doi:10.1371/journal.pone.0296751)
Supplement: S2 Table — Notes: * p<0.1; ** p<0.05; *** p<0.01. Sample weights are applied in the regression. Values in parentheses are standard errors. ICC represents the intraclass correlation, calculated as Σ/(γ+Σ), where Σ is the random intercept variance and γ is the residual variance (i.e., π2/3). AIC and BIC represent Akaike’s and Schwarz’s Bayesian information criteria, respectively. A level of difference greater than 10 in BIC or AIC values between two models suggest that the model with the lower value is favored (Claeskens & Hjort, 2008). Model 3 with the lowest AIC and BIC values is selected for further analysis. (PDF) [file pone.0296751.s009.pdf]

**Table S2.** Estimation of multilevel mixed-effects logistic model on whether out-migrants sending remittances.

Notes: * p<0.1; ** p<0.05; *** p<0.01. Sample weights are applied in the regression. Values in parentheses are standard errors. ICC represents the intraclass correlation, calculated as $\Sigma/(\gamma+\Sigma)$, where $\Sigma$ is the random intercept variance and $\gamma$ is the residual variance (i.e., $\pi^2/3$). AIC and BIC represent Akaike's and Schwarz's Bayesian information criteria, respectively. A level of difference greater than 10 in BIC or AIC values between two models suggest that the model with the lower value is favored (Claeskens & Hjort, 2008). Model 3 with the lowest AIC and BIC values is selected for further analysis.

| **Variable** | **Model 1** | **Model 2** | **Model 3** | **Model 4** |
|---|---|---|---|---|
| CCFP | 0.143** | 0.175** | 0.202** | 0.200** |
| | (0.070) | (0.079) | (0.082) | (0.082) |
| Gender | | 0.182 | 0.257 | 0.258 |
| | | (0.549) | (0.408) | (0.408) |
| Age | | 0.084*** | 0.097*** | 0.097*** |
| | | (0.024) | (0.026) | (0.026) |
| Education | | 0.085 | 0.145 | 0.146 |
| | | (0.092) | (0.081) | (0.081) |
| Province | | 0.602 | 0.682** | 0.682** |
| | | (0.457) | (0.343) | (0.344) |
| Female head | | | -1.648** | -1.663** |
| | | | (0.784) | (0.784) |
| Head age | | | 0.008 | 0.008 |
| | | | (0.017) | (0.017) |
| Head education | | | -0.017 | -0.017 |
| | | | (0.060) | (0.060) |
| Child | | | 0.338 | 0.340 |
| | | | (0.371) | (0.371) |
| Elderly | | | 0.107 | 0.110 |
| | | | (0.258) | (0.258) |
| Elevation | | | -0.683** | -0.694*** |
| | | | (0.264) | (0.261) |
| Slope | | | 0.031 | 0.031 |
| | | | (0.044) | (0.043) |
| Walk | | | 0.033 | 0.033 |
| | | | (0.019) | (0.019) |
| Cropland | | | -0.165 | -0.165 |
| | | | (0.090) | (0.090) |
| Abandonment | | | 0.117 | 0.115 |
| | | | (0.120) | (0.120) |
| Fuelwood | | | 0.035 | 0.035 |
| | | | (0.026) | (0.026) |
| Animal | | | 0.214 | 0.207 |
| | | | (0.333) | (0.334) |
| Business | | | 0.675 | 0.674 |
| | | | (0.566) | (0.568) |
| Off-farm | | | 0.335 | 0.335 |
| | | | (0.409) | (0.409) |
| House | | | 0.356*** | 0.356*** |
| | | | (0.116) | (0.116) |
| Tool | | | 0.261** | 0.262** |
| | | | (0.105) | (0.105) |
| Transportation | | | -0.274 | -0.274 |
| | | | (0.174) | (0.174) |

| | | | | |
|---|---|---|---|---|
| Group size | | | | 0.004 |
| | | | | (0.013) |
| College | | | | -0.064 |
| | | | | (0.048) |
| Hospital | | | | -0.007 |
| | | | | (0.015) |
| School | | | | 0.021 |
| | | | | (0.016) |
| Study site | -1.419*** | -1.037*** | 0.665 | 0.703 |
| | (0.178) | (0.234) | (1.067) | (1.482) |
| Migration years | 0.082** | 0.047 | 0.040 | 0.040 |
| | (0.036) | (0.037) | (0.031) | (0.031) |
| Constant | -0.697*** | -4.318** | -2.677 | -2.829 |
| | (0.237) | (1.563) | (2.100) | (2.072) |
| Variance (constant) | 1.821 | 2.110 | 3.065 | 2.795 |
| | (0.850) | (1.035) | (1.690) | (1.437) |
| ICC | 0.281 | 0.295 | 0.482 | 0.459 |
| | (0.802) | (0.780) | (0.138) | (0.128) |
| Log pseudolikelihood | -5324.09 | -4922.95 | -4326.82 | -4323.59 |
| Wald $Chi^2(1)$ | 117.58*** | 148.21*** | 531.69*** | 570.61*** |
| AIC | 10658.19 | 9863.89 | 8705.63 | 8707.17 |
| BIC | 10681.40 | 9905.68 | 8826.34 | 8846.45 |

## Reference

Claeskens G, Hjort NL. Model selection and model averaging. Cambridge University Press; 2008. Available: https://doi.org/10.1017/CBO9780511790485
