# Supplementary material for: Remittance from migrants reinforces forest recovery for China’s reforestation policy
Source: PLoS One. 2024 Jun 26;19(6):e0296751. doi: 10.1371/journal.pone.0296751 (PMC11207146; doi:10.1371/journal.pone.0296751)
Supplement: S3 Table — Notes: * p<0.1; ** p<0.05; *** p<0.01. Sample weights are applied in the regression. Values in parentheses are standard errors. ICC represents intraclass correlation, calculated as στ2/(γ+στ2) where στ2 is the random intercept variance and γ is the residual variance. AIC and BIC represent Akaike’s and Schwarz’s Bayesian information criteria, respectively. A level of difference greater than 10 in BIC or AIC values between two models suggest that the model with the lower value is favored (Claeskens & Hjort, 2008). Model 3 with the lowest AIC and BIC values is selected for further analysis. (PDF) [file pone.0296751.s010.pdf]

Table S3. Estimation of multilevel mixed-effects model on the amount of remittances sent by out-migrants.

Notes: * p<0.1; ** p<0.05; *** p<0.01. Sample weights are applied in the regression. Values in parentheses are standard errors. ICC represents intraclass correlation, calculated as $\sigma_\tau^2/(\gamma+\sigma_\tau^2)$ where σ_τ^2 is the random intercept variance and γ is the residual variance. AIC and BIC represent Akaike's and Schwarz's Bayesian information criteria, respectively. A level of difference greater than 10 in BIC or AIC values between two models suggest that the model with the lower value is favored (Claeskens & Hjort, 2008). Model 3 with the lowest AIC and BIC values is selected for further analysis.

**Variable**	**Model 1**	**Model 2**	**Model 3**	**Model 4**
CCFP	0.069***	0.082***	0.099***	0.099***
	(0.027)	(0.031)	(0.026)	(0.025)
Gender		-0.144	-0.089	-0.089
		(0.283)	(0.236)	(0.236)
Age		0.059***	0.057***	0.057***
		(0.017)	(0.015)	(0.015)
Education		0.044	0.047	0.047
		(0.046)	(0.035)	(0.035)
Province		0.493**	0.587***	0.588***
		(0.223)	(0.188)	(0.189)
Female head			-0.454	-0.457
			(0.301)	(0.302)
Head age			0.003	0.003
			(0.009)	(0.009)
Head education			-0.006	-0.006
			(0.040)	(0.040)
Child			0.183	0.183
			(0.300)	(0.301)
Elderly			-0.020	-0.019
			(0.158)	(0.159)
Elevation			-0.292***	-0.294***
			(0.086)	(0.088)
Slope			0.020	0.020
			(0.025)	(0.025)
Walk			0.014	0.014
			(0.007)	(0.007)
Cropland			-0.043	-0.043
			(0.027)	(0.027)
Abandonment			0.035	0.035
			(0.087)	(0.088)
Fuelwood			0.015	0.015
			(0.018)	(0.018)
Animal			0.080	0.078
			(0.169)	(0.170)
Business			0.305	0.305
			(0.310)	(0.311)
Off-farm			0.148	0.148
			(0.204)	(0.204)
House			0.255***	0.255***
			(0.079)	(0.079)
Tool			0.134**	0.134**
			(0.055)	(0.056)
Transportation			-0.091	-0.091
			(0.084)	(0.084)

Group size				0.002
				(0.004)
College				-0.008
				(0.010)
Hospital				0.002
				(0.006)
School				0.002
				(0.005)
Study site	-1.323***	-1.009***	0.675	0.633
	(0.158)	(0.154)	(0.482)	(0.573)
Migration years	0.057**	0.019	0.015	0.015
	(0.027)	(0.029)	(0.027)	(0.027)
Constant	1.394***	-0.812	-0.351	-0.422
	(0.169)	(0.790)	(0.973)	(1.022)
Variance (constant)	0.286	0.239	0.302	0.299
	(0.080)	(0.064)	(0.097)	(0.095)
Variance (Residual)	2.462	2.193	1.955	1.954
	(0.277)	(0.218)	(0.219)	(0.219)
ICC	0.104	0.098	0.134	0.133
	(0.028)	(0.024)	(0.041)	(0.040)
Log pseudolikelihood	-19882.79	-19267.77	-18668.37	-18667.86
Wald $Chi^2(1)$	101.29***	170.35***	1333.10***	1604.97***
AIC	39777.58	38555.54	37390.73	37397.72
BIC	39805.43	38601.96	37516.08	37541.63

Reference

Claeskens G, Hjort NL. Model selection and model averaging. Cambridge University Press; 2008. Available: https://doi.org/10.1017/CBO9780511790485
