# Supplementary material for: Remittance from migrants reinforces forest recovery for China’s reforestation policy
Source: PLoS One. 2024 Jun 26;19(6):e0296751. doi: 10.1371/journal.pone.0296751 (PMC11207146; doi:10.1371/journal.pone.0296751)
Supplement: S4 Table — (PDF) [file pone.0296751.s011.pdf]

Table S4. Marginal effects of explanatory variables for multilevel mixed-effects logistic modeling on whether out-migrants sending remittances based on Model-3 (Explanatory variables: CCFP + individual attributes + household characteristics).

Variable	Marginal effect	Standard error	z	p>|z|	95% confidence interval	
CCFP	0.024	0.008	3.05	**0.002**	0.009	0.040
Gender	0.031	0.050	0.62	0.534	-0.066	0.128
Age	0.012	0.003	4.21	**0.000**	0.006	0.017
Education	0.017	0.009	2.01	**0.045**	0.000	0.034
Province	0.082	0.041	2.02	**0.043**	0.002	0.161
Female head	-0.198	0.090	-2.19	**0.028**	-0.375	-0.021
Head age	0.001	0.002	0.48	0.630	-0.003	0.005
Head education	-0.002	0.007	-0.29	0.771	-0.016	0.012
Child	0.041	0.045	0.91	0.363	-0.047	0.128
Elderly	0.013	0.031	0.42	0.675	-0.047	0.073
Elevation	-0.082	0.029	-2.83	**0.005**	-0.139	-0.025
Slope	0.004	0.005	0.74	0.459	-0.006	0.014
Walk	0.004	0.002	1.62	0.104	-0.001	0.009
Cropland	-0.020	0.009	-2.17	**0.030**	-0.038	-0.002
Abandonment	0.014	0.014	1.01	0.314	-0.013	0.041
Fuelwood	0.004	0.003	1.41	0.160	-0.002	0.010
Animal	0.026	0.040	0.63	0.526	-0.054	0.105
Business	0.081	0.063	1.28	0.201	-0.043	0.205
Off-farm	0.040	0.047	0.86	0.389	-0.051	0.132
House	0.043	0.013	3.25	**0.001**	0.017	0.069
Tool	0.031	0.013	2.51	**0.012**	0.007	0.056
Transportation	-0.033	0.019	-1.74	**0.083**	-0.070	0.004
Study site (0=TTZ)	0.079	0.127	0.62	0.533	-0.170	0.329
Migration years	0.005	0.004	1.24	0.216	-0.003	0.012
