# Supplementary material for: Remittance from migrants reinforces forest recovery for China’s reforestation policy
Source: PLoS One. 2024 Jun 26;19(6):e0296751. doi: 10.1371/journal.pone.0296751 (PMC11207146; doi:10.1371/journal.pone.0296751)
Supplement: S5 Table — (PDF) [file pone.0296751.s012.pdf]

Table S5. Marginal effects of explanatory variables for multilevel mixed-effects linear modeling on amounts of remittances sent by out-migrants based on Model-3 (Explanatory variables: CCFP + individual attributes + household characteristics).

**Variable**	**Marginal effect**	**Standard error**	**z**	**p>|z|**	**95% confidence interval**	
CCFP	0.099	0.026	3.86	**0.000**	0.049	0.150
Gender	-0.089	0.236	-0.38	0.706	-0.551	0.373
Age	0.057	0.015	3.74	**0.000**	0.027	0.087
Education	0.047	0.035	1.34	0.182	-0.022	0.116
Province	0.587	0.188	3.12	**0.002**	0.218	0.956
Female head	-0.454	0.301	-1.51	0.132	-1.044	0.137
Head age	0.003	0.009	0.34	0.736	-0.014	0.020
Head education	-0.006	0.040	-0.15	0.881	-0.084	0.072
Child	0.183	0.300	0.61	0.542	-0.405	0.771
Elderly	-0.020	0.158	-0.12	0.901	-0.330	0.290
Elevation	-0.292	0.086	-3.38	**0.001**	-0.461	-0.122
Slope	0.020	0.025	0.82	0.409	-0.028	0.069
Walk	0.014	0.007	1.94	**0.053**	0.000	0.028
Cropland	-0.043	0.027	-1.61	0.106	-0.096	0.009
Abandonment	0.035	0.087	0.40	0.687	-0.136	0.207
Fuelwood	0.015	0.018	0.80	0.424	-0.021	0.051
Animal	0.080	0.169	0.47	0.635	-0.251	0.411
Business	0.305	0.310	0.98	0.325	-0.302	0.912
Off-farm	0.148	0.204	0.73	0.468	-0.252	0.547
House	0.255	0.079	3.22	**0.001**	0.100	0.411
Tool	0.134	0.055	2.42	**0.016**	0.025	0.243
Transportation	-0.091	0.084	-1.08	0.282	-0.256	0.075
Study site (0=TTZ)	0.675	0.482	1.40	0.161	-0.269	1.620
Migration years	0.015	0.027	0.54	0.592	-0.039	0.068
