# Supplementary material for: Remittance from migrants reinforces forest recovery for China’s reforestation policy
Source: PLoS One. 2024 Jun 26;19(6):e0296751. doi: 10.1371/journal.pone.0296751 (PMC11207146; doi:10.1371/journal.pone.0296751)
Supplement: S6 Table — Notes: mu measures the unit management size of land in China; 1 mu = 666.7 m2. Scores of house condition, farm tools and transportation evaluate the physical capital endowment of a household reflecting the wellness status (Song et al. 2018) [39]. Values are the absolute proportion differences for binary variables and absolute standard mean differences for continuous variables. TTC represents the Tiantangzhai site and J&C represents the Jichang and Checheng site. (PDF) [file pone.0296751.s013.pdf]

Table S6. Selection of variables for matching households receiving remittances with households not receiving remittances.

Notes: mu measures the unit management size of land in China; 1 mu = 666.7 m^2. Scores of house condition, farm tools and transportation evaluate the physical capital endowment of a household reflecting the wellness status (Song et al. 2018). Values are the absolute proportion differences for binary variables and absolute standard mean differences for continuous variables. TTC represents the Tiantangzhai site and J&C represents the Jichang and Checheng site.

**Variable**	**Difference between treated and control groups**	
	**Before match**	**After match**
General context indicated by a dummy variable for different study sites (0=TTZ, 1=J&C)	0.381	0.004
Slope at house location (degree)	0.150	0.049
Total amount of cropland managed by household (mu)	-0.201	-0.146
Whether engaging off-farm activities within county boundary (0=no, 1=yes)	0.029	-0.008
Score of house condition	-0.434	0.101
Score of transportation tools	0.222	-0.026

Reference

Song C, Bilsborrow R, Jagger P, Zhang Q, Chen X, Huang Q. Rural household energy use and its determinants in China: How important are influences of payment for ecosystem services vs. other factors? Ecological Economics. 2018;145: 148–159. doi:10.1016/j.ecolecon.2017.08.028
