# Supplementary material for: Remittance from migrants reinforces forest recovery for China’s reforestation policy
Source: PLoS One. 2024 Jun 26;19(6):e0296751. doi: 10.1371/journal.pone.0296751 (PMC11207146; doi:10.1371/journal.pone.0296751)
Supplement: S1 Text — (PDF) [file pone.0296751.s015.pdf]

Supplementary Text S1

Description of study sites

The first site, Jichang & Checheng (J&C), is located on the Loess Plateau within a geospatial extent of 110°34′E - 110°57′E and 36°3′N - 36°16′N (489 km^2) in southwestern Shanxi Province in northern China (Fig. S1, upper right). The landform is characterized by the Loess Plateau, also known as the "Yellow Earth Plateau", with elevation ranging from 830 m to 1,820 m above the mean sea level. The climate in this region is semi-arid with Asian monsoon providing limited moisture in the summer. The mean annual temperature is 10°C, and the mean annual precipitation is 544 mm (Song et al. 2014), the majority of which falls during June to September. Both natural forests and agroforests (e.g., apple or walnut orchards) comprise nearly 60% of the total area of the townships. There are around 6,000 households living in 195 resident groups. Dryland is the dominant cropland type. Despite relatively limited economic opportunities, residents pursue diverse economic activities, including orchard cultivation, livestock husbandry, labor out- migration, and local off-farm employment.

The second site, Tiantangzhai (TTZ), spans a geospatial extent of 115°39′E - 115°53′E and 31°8′N - 31°17′N (189 km^2) in the Dabieshan mountain ranges along the western Anhui, border Hebei in central-eastern China (Fig. S1, the lower right panel). This region lies in the subtropical climate zone with a mean annual temperature of 16.4°C and a mean annual total precipitation of 1,350 mm. The elevation ranges from 362 to 1,729 m above the mean sea level. Tiantangzhai forms the core of Tianma National Nature Reserve, which was designated in the early 1990s to protect the last remaining patches of secondary evergreen broadleaf forests in eastern China (Han et al. 2011). The landscape is dominated by natural forests, which cover about two thirds of the total area of TTZ. The township consists of seven administrative villages, with over 4,000 households distributed in 165 resident groups. Local residents live primarily on growing paddy rice and dryland crops such as corn, but they also conduct a variety of other livelihood activities, similar to the J&C site, such as raising livestock (e.g., pigs and chickens), extracting forest resources (e.g., fuelwood), sending out labor migrants, and occasionally establishing small local businesses.

Initial enrollment of cropland to the Conversion of Cropland to Forest Program (CCFP) started in 2002 at both study sites. As in other CCFP areas, the governmental agencies offered technical assistance for participating households to establish forest stands on targeted cropland parcels that are prone to soil erosion (Bennett et al. 2014). Households who enrolled qualified cropland parcels in the CCFP would receive payments directly from the central government annually as long as the planted trees on their enrolled cropland survive through the year. The payment amount depends exclusively on the total area of cropland enrolled, while the payment rate differs between the two study sites considering the different opportunity costs of forgoing farming the cropland. The annual payment rates are 90 Yuan/mu in J&C and 125 Yuan/mu in TTZ (100 Yuan = US$12.20 in 2002; 1 mu = 666.67 m^2), respectively. Most households maintained their enrollment in the program in the survey years, i.e., 2014 in TTZ and 2015 in J&C. Major tree species planted under the CCFP include black locust (*Robinia pseudoacacia*) and walnut (*Juglans regia*) in J&C and Maple (*Acer saccharum*) in TTZ. During the

implementation of the CCFP, both study sites witnessed labor out-migration as a critical livelihood strategy, expecting remittance in return. According to the household surveys, about 38% and 52% of the individuals aged 16-59 in samples had migrated out for at least 6 consecutive months and remained away at the time of the interview in J&C and TTZ, respectively. Of these migrants, 66% (J&C) and 85% (TTZ) migrated outside the local counties to distant cities for better job opportunities. Despite the large volumes of rural-to-urban migration, origin households that are dependent on croplands and forests may or may not change their main livelihood strategies. A previous study found that the CCFP payments significantly influenced migration decisions and subsequent forest change surrounding the households, and the effects nevertheless show significant heterogeneity among households (Zhang et al. 2020). What drive this heterogeneous effect is unknown. Here, we hypothesize that remittance, not migration per se, affects forest change surrounding the origin households. We collected data from household and community surveys and obtained satellite images to investigate the mediating role played by remittance sent by migration in forest dynamics. The CCFP features China's efforts of ecological restoration that contributes to forest transition (Li et al. 2019). This study uncovers the mechanism of how migration moderates forest conservation and local livelihoods through the lens of remittance, which is less known in the literature.

References

1. Song C, Zhang Y, Mei Y, Liu H, Zhang Z, Zhang Q, et al. Sustainability of Forests Created by China's Sloping Land Conversion Program: A comparison among three sites in Anhui, Hubei and Shanxi. Forest Policy and Economics. 2014;38: 161–167. doi:10.1016/j.forpol.2013.08.012
2. Han G, Fang W-T, Huang Y-W. Classification and Influential Factors in the Perceived Tourism Impacts of Community Residents on Nature-based Destinations: China's Tiantangzhai Scenic Area. Procedia Environmental Sciences. 2011;10: 2010–2015. doi:10.1016/j.proenv.2011.09.315
3. Bennett M, Xie C, Hogarth N, Peng D, Putzel L. China's conversion of cropland to forest program for household delivery of ecosystem services: How important is a local implementation regime to survival rate outcomes? Forests. 2014;5: 2345–2376. doi:10.3390/f5092345
4. Zhang Q, Wang Y, Tao S, Bilsborrow RE, Qiu T, Liu C, et al. Divergent socioeconomic-ecological outcomes of China's conversion of cropland to forest program in the subtropical mountainous area and the semi-arid Loess Plateau. Ecosystem Services. 2020;45: 101167. doi:10.1016/j.ecoser.2020.101167
5. Li L, Chhatre A, Liu J. Multiple drivers and pathways to China's forest transition. Forest Policy and Economics. 2019;106: 101962. doi:10.1016/j.forpol.2019.101962
