# Supplementary material for: Remittance from migrants reinforces forest recovery for China’s reforestation policy
Source: PLoS One. 2024 Jun 26;19(6):e0296751. doi: 10.1371/journal.pone.0296751 (PMC11207146; doi:10.1371/journal.pone.0296751)
Supplement: S2 Text — (PDF) [file pone.0296751.s016.pdf]

Supplementary Text S2

Robustness and sensitivity check for multilevel modeling and matching

Telecoupling of migration social network: We recorded the destination of city for each out-migrant in our surveyed households and depicted the migration social network featuring telecoupling relationships based on information of migration and remittance. Through the city-level social network, a major finding is that the remittance amount per migrant does not decline or diminish as the migration distance increase (Fig. 1), supporting the telecoupling framework (Hull & Liu, 2018). This evidence may be weakened by the heterogeneity of economic level at the city scale, i.e., out-migrants purposely move to cities with relatively better opportunities compared to others within a same province. Therefore, we aggregated the information to the provincial level by setting the provincial capital as the destinations. The provincial-level results are found to be consistent to those at the city level, showing that the amount of remittance per migrant does not significantly change among migrants with destinations in different distances (Fig. S2). Specifically, remittance per migrant is slightly higher (albeit statistically insignificant) for longer-distance migrants than shorter-distance migrants. Note that the aim is not to test that the telecoupling relationship increases with distance, but we argue that the long distance does not weaken such a connection, i.e., an out-migrant would be less likely to send remittance if migrating to a farther city. Therefore, the findings remain supportive to our main argument of the existence of telecoupling.

Multilevel analysis of CCFP on remittance: To tested the robustness of the estimated marginal effects in the multilevel modeling, we used weighted bootstrapping (Chernick, 2011) to mimic the weighted sampling processes as adopted in the household surveys (Zhang et al. 2020; Song et al. 2018). The bootstrapping method involves random sampling with replacement from the out-migrant sample. The probability of a single migrant being selected is weighted by the sample weight derived from the household survey (Bilsborrow, 2016). Due to the randomness, we ran modeling for 1,000 times for the bootstrapping and plotted the distribution of all the simulated marginal effects. Results show a high level of consistency of the simulation outcomes based on bootstrapping with the multilevel modeling (Fig. 2, right panels) using the same explanatory variables in Model 3. For modeling whether an out-migrant sends remittance, the averaged marginal effect is 0.027 (min: 0.018, max: 0.038) which is only 0.003 (11.7%) higher than that of the multilevel model (Table S5). For modeling remittance amount, the averaged marginal effect is 0.101 (min: 0.079, max: 0.122) which is only 0.002 (15.1%) higher than that of the multilevel model (Table S6). In both cases, the range of the values are above the zero, suggesting a non-neutral effect of the CCFP on remittance.

Treatment effect of remittance on forest change: In propensity score matching, the estimated treatment effects can be sensitive to the caliper threshold, which limit the difference range for each pair of observation to be matched from control and treated groups (Austin, 2011). A narrower range would generate a pair of observations with closer scores (i.e., a higher level of matching in terms of propensity of receiving remittance in our case), but risk losing observations during matching. We performed sensitivity analysis for a range of caliper threshold from 0.001 to 0.010 at a step of 0.001, which was empirically based on the distribution of score difference

after matching (Fig. S4). We plotted the varying changes of forest dynamics (cover & EVI) for treated and controlled groups and their differences with t-test outcomes (treated/controlled: difference of mean from zero; treatment effect: difference in means between groups). We also tested the same outcomes by setting buffer at various sizes, from 25m to 200m at a step of 25m. To justify the reliability of the statistical tests, the proportion of observations retained at each caliper threshold was obtained. Results show a high consistency level of treatment effects across all caliper thresholds (Fig. S6), suggesting that the estimated effects of remittance on forest change are robust. For instance, the highest level of consistency is observed when setting buffer size at the 75m and 100m of primary interest, where significant outcomes occur when the retained observations reach 80% or more.

References

Austin PC. Optimal caliper widths for propensity-score matching when estimating differences in means and differences in proportions in observational studies. Pharmaceut Statist. 2011;10: 150–161. doi:10.1002/pst.433

Bilsborrow RE. Concepts, definitions and data collection approaches. In: White MJ, editor. International Handbook of Migration and Population Distribution. Dordrecht: Springer Netherlands; 2016. pp. 109–156. doi:10.1007/978-94-017-7282-2_7

Chernick MR. Bootstrap Methods: a Guide for Practitioners and Researchers. Somerset: Wiley; 2011.

Hull V, Liu J. Telecoupling: A new frontier for global sustainability. E&S. 2018;23: art41. doi:10.5751/ES-10494-230441

Song C, Bilsborrow R, Jagger P, Zhang Q, Chen X, Huang Q. Rural household energy use and its determinants in China: How important are influences of payment for ecosystem services vs. other factors? Ecological Economics. 2018;145: 148–159. doi:10.1016/j.ecolecon.2017.08.028

Zhang Q, Wang Y, Tao S, Bilsborrow RE, Qiu T, Liu C, et al. Divergent socioeconomic-ecological outcomes of China's conversion of cropland to forest program in the subtropical mountainous area and the semi-arid Loess Plateau. Ecosystem Services. 2020;45: 101167. doi:10.1016/j.ecoser.2020.101167
